# *PathME*: pathway based multi-modal sparse autoencoders for clustering of patient-level multi-omics data

**DOI:** 10.1186/s12859-020-3465-2

**Published:** 2020-04-16

**Authors:** Amina Lemsara, Salima Ouadfel, Holger Fröhlich

**Affiliations:** 1 Computer Science Department, University of Constantine 2, 25016 Constantine, Algeria; 2 University of Bonn, Bonn-Aachen, International Center for IT, 53115 Bonn, Germany; 3 Fraunhofer Institute for, Algorithms and Scientific, Computing (SCAI), 53754 Sankt, Augustin Germany

**Keywords:** Deep learning, Patient clustering, Multi-omics

## Abstract

**Background:**

Recent years have witnessed an increasing interest in multi-omics data, because these data allow for better understanding complex diseases such as cancer on a molecular system level. In addition, multi-omics data increase the chance to robustly identify molecular patient sub-groups and hence open the door towards a better personalized treatment of diseases. Several methods have been proposed for unsupervised clustering of multi-omics data. However, a number of challenges remain, such as the magnitude of features and the large difference in dimensionality across different omics data sources.

**Results:**

We propose a multi-modal sparse denoising autoencoder framework coupled with sparse non-negative matrix factorization to robustly cluster patients based on multi-omics data. The proposed model specifically leverages pathway information to effectively reduce the dimensionality of omics data into a pathway and patient specific score profile. In consequence, our method allows us to understand, which pathway is a feature of which particular patient cluster. Moreover, recently proposed machine learning techniques allow us to disentangle the specific impact of each individual omics feature on a pathway score. We applied our method to cluster patients in several cancer datasets using gene expression, miRNA expression, DNA methylation and CNVs, demonstrating the possibility to obtain biologically plausible disease subtypes characterized by specific molecular features. Comparison against several competing methods showed a competitive clustering performance. In addition, post-hoc analysis of somatic mutations and clinical data provided supporting evidence and interpretation of the identified clusters.

**Conclusions:**

Our suggested multi-modal sparse denoising autoencoder approach allows for an effective and interpretable integration of multi-omics data on pathway level while addressing the high dimensional character of omics data. Patient specific pathway score profiles derived from our model allow for a robust identification of disease subgroups.

## Background

Precision medicine aims for the delivery of the right treatment for the right patients. One important goal is therefore to identify molecular sub-types of diseases, which opens the opportunity for a better targeted therapy of malignancies in the future. In that context high throughput omics data has been extensively used. Prominent examples include the gene expression based groupings of breast cancer and glioblastoma multiforme (GBM) into four clusters [1, 2]. However, analysis of one type of omics data alone provides only a very limited view on a complex and systemic disease such as cancer. Correspondingly, parallel analysis of multiple omics data types is needed and employed more and more routinely [3–5]. However, leveraging the full potential of multi-omics data requires statistical data fusion, which comes along with a number of unique challenges, including differences in data types (e.g. numerical vs discrete), scale, data quality and dimension (e.g. hundreds of thousands of SNPs vs few hundred miRNAs), see Ahmad and Fröhlich for a review [6].

Several authors have proposed methods, which allow for integrating multi-modal omics data into one clustering model and thus allow for detection of clusters that are consistently supported by several biological scales [7–10]. Prominent examples include Similarity Network Fusion (SNF) [11], iCluster [12] and multi-view non-negative matrix factorization [13], see [6] for a more complete overview and discussion. A challenge that all these methods face is the high dimensional character of omics data and the large difference of the number of features across different omics modalities.

In this paper we propose an unsupervised multi-modal neural network architecture to learn a low dimensional embedding of omics features from multiple sources, which can be mapped to the same biological pathway. The result of such an embedding is one score per pathway and patient. In a second step we then combine scores of multiple pathways into a profile for each patient, which we use to bi-cluster patients and pathways via consensus sparse non-negative matrix factorization (sNMF) [14], hence providing insights into interpretable and cluster specific pathways of distinct patient subgroups. Furthermore, by leveraging a recently proposed machine learning technique [15], we are also able to disentangle the relevance of individual genes, miRNAs, CNVs and CpG sites for each individual patient subgroup.

We demonstrate the utility of our approach in comparison to conventional sNMF based clustering based on a gene expression leukemia dataset, and in comparison to SNF and iCluster based on four cancer datasets from the GDC Data Portal and cBioPortal, respectively. We specifically show the association of clusters identified by our Pathway based Multi-modal autoEncoder (*PathME*) method to well-known somatic mutation patterns and a variety of clinical outcomes, including survival. Moreover, we highlight the possibility to interpret features selected by our method for individual patient subgroups in the light of existing disease knowledge.

## Methods

### Overview

**Fig. 1:**
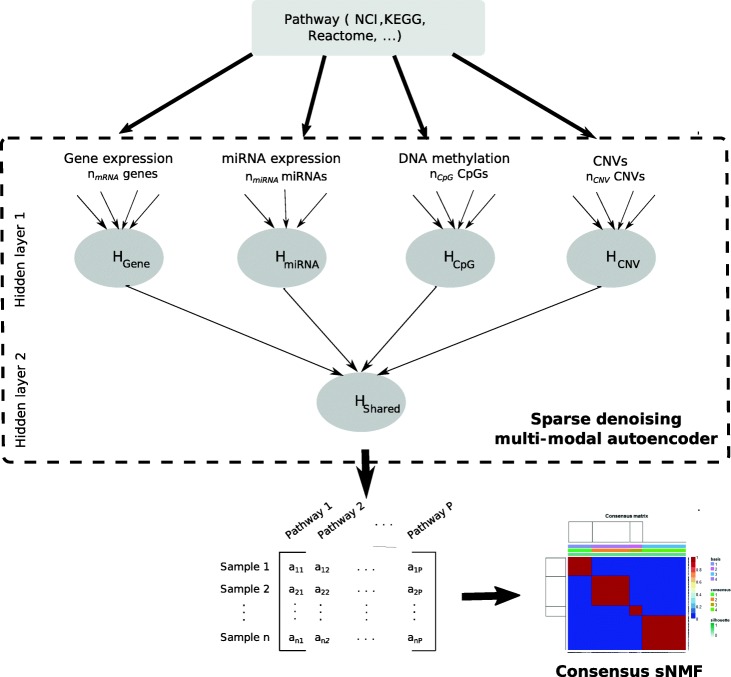
Conceptual overview about our approach: Multi-omics feature mapping to a specific pathway are summarized into a pathway level score via a sparse denoising multi-modal autoencoder architecture. Hidden layer 1 consists of up to [*p*_*j*_/2] hidden units per omics modality, where p_j is the number of features in omics type *j*. Hidden units for omics modality *j* are densely connected to input features of the same omics type, but there are no connections from input features of other data modalities. Hidden layer 2 consists of one hidden unit, which represents the overall multi-omics pathway score. Concatenation of *P* multi-omics pathway scores for each patient allows for application of consensus sparse NMF clustering in a subsequent step

The overall aim of our proposed multi-modal autoencoder framework is to embed *k* patient-level omics data types mapping to a particular pathway of interest into a common low dimensional latent space. Our method thus compresses hundreds of original features into one score per pathway and patient. Conducting the same embedding for *P* pathways results into a pathway profile representation, which we use to stratify patients based on sparse NMF in an unsupervised manner [14]. This effectively allows for a bi-clustering of patients and pathways and thus ensures a certain level of interpretation. Overall our proposed *PathME* method consists of four major steps (Fig. 1):

1. Mapping of omics features from each data source to pathways.
2. Estimation of a per-patient score for each pathway using multi-modal sparse denoising autoencoders.
3. Bi-clustering of patients using consensus sNMF.
4. Interpretation of clusters and cluster specific pathway scores using recent statistical and game theoretic methods.

In the following we describe each of these steps in more detail.

### Mapping of Omics features to pathways

To demonstrate the principle of our method in this paper we used combinations of gene expression, miRNA expression, DNA methylation (chip based) and copy number variation (CNV) data. Entrez genes IDs were mapped to NCI pathways [16] using the graphite R-package [17], but of course other pathway databases could be used as well. For DNA methylation data we relied on the annotation by the manifacturer to map individual CpGs to Entrez gene IDs. For assignment of CNVs to genes we relied on the mapping provided by The Cancer Genome Atlas (TCGA), which uses the Genomic Identification of Significant Targets in Cancer (GISTIC2) method [18]. TCGA provides for each patient a list of CNVs mapped to Entrez gene IDs. These are available for download via http://firebrowse.org/. For miRNA data, we considered the predicted miRNA target genes (again as Entrez gene IDs) obtained from miRBase [19]. Overall, CpGs, CNVs and miRNAs were mapped to Entrez gene IDs and Entrez gene IDs to pathways. Hence, all relevant omics modalities considered in this paper could be mapped to pathways.

### Estimation of pathway scores via multi-modal sparse Denoising autoencoders

#### Deep Denoising autoencoders

We start by explaining a standard autoencoder [20]. Briefly, an autoencoder network takes a feature vector *x* ∈ *ℝ* ^*d*^ as input and transforms / encodes it to a hidden representation *y* ∈ *ℝ* ^*q*^ (typically *q < d*) via

1 $$ y=s\ \left( Wx+b\right)\Big) $$

where *s*(·) is a non-linear activation function, e.g. tanh or rectified linear unit. Matrix *W* consists of weights and *b* is a bias vector. Several encoding steps can be performed sequentially, resulting into a deep encoder.

The latent representation *y* can be decoded / mapped back via

2 $$ z=s\left({W}^0y+{b}^0\right) $$

where *W*^0^*,b*^0^ are other weights and a bias vector. Again, several decoding steps can be performed sequentially, resulting into a deep decoder. Encoder plus decoder network together form a (deep) autoencoder. In most cases (as well as in our work) the decoder network has a laterally reversed architecture to the encoder. That means the input to the encoder network has the same number of units as the last layer of the decoder (which is at the same time the output of the entire autoencoder). Hidden layer 1 in the encoder has the same architecture as the second last layer of the decoder, and so on.

The objective of training a deep autoencoder is to minimize the reconstruction loss *L*(·*,*·) (e.g. the mean squared difference between original input *x* and reconstructed input, i.e. output of the autoencoder, *z*) by updating weights *W,W*^0^ and biases *b,b*^0^:

3 $$ argmi{n}_{W,{W}^0,b,{b}^0}L\left(x,z\right) $$

The training can be conducted via common stochastic gradient descent methods.

To further increase robustness of the representation learning Vincent et al. [21] suggested to add random noise to input features while attempting to reconstruct original data, which results into a denoising autoencoder.

#### Multi-modal Denoising autoencoders

In our case we have multiple omics data sources *X*_1_*,X*_2_*,*···*,X*_*k*_ from the same *n* patients mapping to a particular pathway of interest. To account for this multimodal character of the data we suggest the architecture shown in Fig. 1: Each data modality *j* is first encoded separately into up to *p*_*j*_*/*2 hidden units (using dense connections between inputs and hidden units), where *p*_*j*_ is the number of input features for omics type *j*. In Fig. 1 these units in the first hidden layer are denoted as *H*_*gene*_, *H*_*miRNA*_, etc. The exact number of hidden units for each data modality is determined via a hyper-parameter optimization, which is described in Section 1.3.4. In a second step units from the first hidden layer are further encoded (via dense connections) into one pathway score, which is denoted as *H*_*shared*_. *H*_*shared*_ is the only unit in the second hidden layer. As mentioned before, the decoder has a laterally reversed architecture to the encoder and is thus not explicitly shown in Fig. 1.

Note that our suggested architecture has significantly fewer trainable weights than a fully connected standard autoencoder network, because initially a separate hidden representation within each data modality is learned.

The specific architecture of our multi-modal (denoising) autoencoder induces a modified loss function for training the network compared to a standard autoencoder.

More specifically, let *W* denote the set of all trainable network parameters. Let ${h}_W\left({x}_j^{(i)}\right)$ denote the reconstruction of feature vector *x*^(*i*)^ corresponding to patient *i* in data modality *j* at the output layer of the autoencoder. Our training objective is then to minimize

4 $$ \sigma (W)=\frac{1}{n}\sum \limits_{j=1}^k\sum \limits_{i=1}^n{\left\Vert {h}_W\left({x}_j^{(i)}\right)-{x}_j^{(i)}\right\Vert}^2 $$

That means we aim to find network parameters *W*, which jointly minimize the reconstruction error of features within each data modality.

#### Introducing Sparsity

To control overfitting, we regularize our model by enforcing sparsity of weights *W*. More specifically, we used two techniques for this purpose: i) random drop-out of input features with predefined probability *p* [22]; ii) a sparse group lasso penalty for features at the input layer [23]. The sparse group lasso is an extension of the classical lasso algorithm [24], which has originally been introduced in the context of generalized linear models. The sparse group lasso enforces a sparse regression model by jointly pushing coefficients of certain predefined groups of features towards zero, i.e. there is a feature selection at the group level similar to the group lasso [25]. Furthermore, also sparsity within each group of features is promoted.

The idea of the sparse group lasso can also be applied to neural networks. More specifically, by adding a sparse group lasso penalty we modify our training objective as:

5 $$ {\displaystyle \begin{array}{l}F(W)=\frac{1}{2}\sigma (W)\\ {}\kern3.5em +\frac{\lambda }{2}\left(\left(1-\alpha \right)\sum \limits_{l=1}^k\sqrt{s_l\ast {s}_{lsucc}}\sum \limits_{i=1}^{s_l}\sum \limits_{j=1}^{s_{lsucc}}{\left({W}_{ij}^l\right)}^2+\alpha \sum \limits_{l=1}^{d-k}\sum \limits_{i=1}^{s_l}\sum \limits_{j=1}^{s_{lsucc}}|{W}_{ij}^l|\right)\end{array}} $$

where *d* is the number of layers, *s*_*l*_ is the number of units in *l*^*th*^ layer, s_l_succ is the number of units in the successor layer of the *l*^*th*^ layer, *λ* is the weight decay parameter, *α* is a convex combination of the lasso and group lasso penalties. The first penalty in the second line (*l*_2_-norm of weights) promotes sparsity at the group (here: omics modality) level. The second penalty in the third line of Eq. (5) (*l*_1_-norm of weights) promotes sparsity at the individual feature level within each data modality. Parameter *α* ∈ [0*,*1] balances between sparse selection of entire omics data modalities and sparsity within each data modality. If *α* = 1 we recover the original lasso penalty by Tibshirani [24]. If *α* = 0 we get the group lasso penalty by Yuan and Lin [25].

#### Training and optimizing the multi-modal sparse Denoising autoencoder

For training of our muli-modal sparse denoising autoencoder we use mini-batch stochastic gradient descent [26, 27], i.e. the training procedure updates parameters iteratively using randomly chosen small mini-batches of patients instead of the entire training set. We evaluated different variants of the stochastic gradient descent algorithm: momentum [28], RMSProp [29], adam [30] and Nesterov-accelerated Adam (nadam) [31]. To account for differences in numerical ranges of data modalities we employed batch normalization of hidden units [32], i.e. scaled their inputs to mean zero and standard deviation one. The complementary batch de-normalization was performed at the output layer. Note that batch normalization also accounts for the covariance shift of network weights and typically yields a dramatic speed-up of the training procedure [32].

All experiments in this paper were carried out with tanh activation functions, because initial results looked most promising. In addition to the regularization techniques described in last paragraph we incorporated an early stopping mechanism in the training process [26]. More specifically, we stopped training when there was no improvement in the loss function for more than twenty iterations.

Altogether we considered the following hyper-parameters for training or model:

1. mini-batch size ∈ {4*,*8*,*16*,*32}
2. sparse group lasso parameters *α* ∈ [0*,*1] and *λ* ∈ [10^− 9^*,*···*,*10^− 1^]
3. learning rate *ρ* ∈ [10^− 5^*,*···*,*10^− 1^]
4. probability *p* of dropout of an input feature *p* ∈ [0*.*5*,*1]
5. number of units in the first hidden layer in the range [1*,*···*,p*_*j*_*/*2], where *p*_*j*_ is the input size of the *j*^*th*^ omics modality. Note that we used for the decoder network an architecture that was laterally reversed to the encoder.

To deal with the large number of different hyper-parameters we used Bayesian hyper-parameter optimization with 50 evaluations [33]. Within this procedure each selected candidate set of hyper-parameters was assessed via the reconstruction error of the model on unseen test data via a 5-fold cross-validation procedure. That means we randomly split the entire dataset into 5-folds and sequentially left out one of these folds (i.e. 20% of the data) for validation / testing, whereas the rest of the data was used to learn network weights. The entire implementation has been conducted in TensorFlow and is available in the Supplementary files to this paper.

### Patient bi-clustering via consensus sparse non-negative matrix factorization

Our suggested multi-modal sparse autoencoder resulted into a patient specific pathway score profile. We used this matrix to jointly identify clusters of patients and cluster specific pathways. We employed sparse non-negative matrix factorization (sNMF) for this purpose [14], which is an extension of the algorithm by Lee et al. [34]. Briefly, this algorithm factorizes a data matrix *X* (here: *P* pathways ×*n* patients) as *X* ≈ *BH*, where *B* is a non-negative *P* × *m* matrix containing basis vectors and *H* is a non-negative *m* × *n* matrix containing coefficient vectors. The solution is found by minimizing the reconstruction error between *X* and *BH* via multiplicative updates. Sparse NMF extends this approach by additionally enforcing sparsity of matrix *B*:

6 $$ {\mathit{\min}}_{B,H}\frac{1}{2}\left\{{\left\Vert X- BH\right\Vert}_F^2+\eta {\left\Vert B\right\Vert}_F^2+\beta \sum \limits_{j=1}^n{\left\Vert H\left(:,j\right)\right\Vert}_1^2\right\},s.t.B,H\ge 0 $$

where *η >* 0 and *β >* 0 are regularization parameters, and *H*(:*,j*) is the *j*th column vector of *H*. According to Kim et al. [14] *η* was set to the maximum value of *X*. Sparse NMF effectively yields a bi-clustering of patients and pathways and thus allows for identifying a cluster specific pathway profile. For doing so we employed the method by Carmona et al. [35]: For each basis component *i* (i.e. *i*th column in *B*) values are first sorted by decreasing magnitude. Then, only the first consecutive features are considered as most descriptive for cluster *i*. That means *PathME* allows for assessing the most descriptive pathways per data modality (e.g. gene expression, CNVs) and cluster.

To help the interpretation of these most descriptive pathways we calculated the fraction of genes carrying any form of somatic mutation, resulting into a mutational burden score. We then compared mutational burden across clusters via a Kruskal-Wallis test. *P*-values were corrected for multiple testing via the Benjamini-Hochberg method [36].

NMF based matrix factorization is performed in an iterative fashion and starting from some initial conditions. The obtained solution is thus sensitive to the initialization. To account for this aspect we performed 500 repeated runs of the entire sNMF algorithm, starting from different random initializations of *B* and *H* [14], as implemented in the R-package NMF [37]. One possible strategy is then to look for the individual solutions yielding minimal reconstruction loss, which resembles the approach typically taken in k-means clustering. Another approach, which we followed here, is to conduct consensus clustering over these 500 individual clustering solutions by hierarchical clustering of the consensus matrix [38]. The agreement of the hierarchical clustering with the similarity of patients according to the consensus matrix can be assessed via the cophenetic correlation [39]. The cophenetic correlation can then be used for model selection. More specifically, we here used this measure to tune the regularization beta in the range [0*.*001*,*1] and to find the optimal rank of the factorization (i.e. number of clusters) in the range 2*,...,*9. While doing so we aimed for comparing the cophenetic correlation achieved by our consensus sNMF algorithm with the cophenetic correlation achieved by chance. Since consensus sNMF clustering is computationally quite costly (as it is the result of 500 sparse non-negative matrix factorizations) we limited ourselves to 40 random permutations of the data matrix *X* here. For each of these random permutations we re-ran the entire consensus sNMF algorithm. We then looked for the smallest number of clusters *m*, for which the cophenetic correlation achieved on the basis of the original data exceeded the upper bound of the 95% confidence interval achieved on the basis of randomly permuted data. In addition, we recorded also the silhouette index as a widely used distance based clustering index [40]. The silhouette *s*(*i*) for data point (here: patient) *i* assigned to cluster *C*_*i*_ is defined as.

7 $$ s(i)=\frac{b(i)-a(i)}{\max\;\left(a(i),b(i)\right)} $$

provided that |*C*_*i*_| *>* 1, and 0 otherwise. Furthermore, *a*(*i*) is the mean distance of data point *i* to all other data points in cluster *C*_*i*_, and *b*(*i*) is defined as.

8 $$ b(i)=\underset{k\ne i}{\min}\frac{1}{\left|{C}_k\right|}\sum \limits_{j\in {C}_k}d\left(i,j\right) $$

where *d*(*i,j*) is the distance between data points *i* and *j*. The silhouette *s*(*i*) ranges from − 1 to 1, where 0 indicates that data point *i* falls in between two clusters. The *silhouette index* is defined as the mean *s*(*i*) over all data points. A value closer to 1 indicates a more tightly grouping of data points into clusters.

#### Interpretation of cluster specific pathway scores via SHAP

One of the main criticism of neural network based approaches (here: autoencoders) is the difficulty to interpret them. Recently, Lundberg et al. [15] proposed a game theoretic framework to address this issue. Briefly, the idea behind Shapley Additive exPlanations (SHAP) is that the relevance of feature *i* for model output *f*(*x*) can be regarded as the average weighted difference between *f*(*x*) and outputs from all possible models trained on subsets of features, excluding feature *i*:

9 $$ {\phi}_i(x)\sum \limits_{S\subseteq F}\frac{\mid S\mid !\left(|F|-|S|-1\right)}{F}\left(f\left({x}_S\right)-f\left({x}_{S\setminus i}\right)\right) $$

where *F* is the set of all features. The authors propose several local approximation techniques, which can circumvent the exact combinatorial calculation of Φ_*i*_(*x*), one which is specifically tailored towards neural networks (Deep SHAP). Deep SHAP effectively combines SHAP values calculated for smaller components of a neural network into SHAP values for the entire network. We refer to Lundberg et al. [15] for details. In this work we used SHAP to understand the impact of individual omics modalities and features on the autoencoded score that we learned for each pathway. SHAP results into a patient specific score that may be positive (feature *i* increases *f*(*x*)) or negative (feature *i* decreases *f*(*x*)). In agreement to [15] we considered the mean of the absolute SHAP values per omics feature to score the overall impact of a variable on the pathway score. Moreover, we investigated the overall mean of absolute SHAP values per omics data modality. Hence, our *PathME* method allows for interpreting the influence of pathways, omics data modalities and individual markers on dedicated patient sub-groups.

#### Association to clinical features

An important question is, in how far molecular subgroups might be clinically relevant. For this purpose we investigated for each cancer type a wide range of available clinical features for each disease, including different survival endpoints. More specifically, we considered overall survival (OS), progression free survival (PFS) and disease free survival (DFS), as defined in Liu et al. [41]. We first checked nominal significance of the association to age via a Cox regression model that only contained age as predictor. If this was true, we fitted a Cox regression model that contained a factor “cluster” plus age as predictors. This model was then compared against the “null” Cox regression model that only contained age as predictor. Both models were compared via a likelihood ratio test, and the corresponding *p*-value was reported as the significance of the age corrected association to the clustering. In case of no significant association with age a conventional log-rank test was used.

Significance of additional categorical clinical variables (e.g. gender) was tested via a *χ*^2^ test, including the agreement with existing molecular classification schemes. For numerical variables a one-way ANOVA was applied.

Multiple testing correction of *p*-values was performed via the Benjamini-Hochberg method to control false discovery rate (FDR) [36].

#### Compared approaches

We compared our *PathME* approach against two competing multi-omics clustering methods: 1) SNF and 2) iCluster. For both methods we performed a pre-filtering of features: We selected only features that mapped to the same pathways used by our method. In addition, for the iCluster method we further down-filtered these features to the 100–200 most variable ones (depending on each disease). This was done to increase the robustness of the iCluster method and get better separated clusters.

According to the suggestion by the respective authors of each method the number of clusters for SNF was selected via the eigen-gap method [11] and for iCluster via the proportion of deviation score [12].

Although we motivated the development of our approach by the use of multi-omics data, *PathME* can in principle also be applied to single omics data. We therefore compared *PathME* also against sNMF directly applied to individual omics features, which could be mapped to NCI pathways.

## Results

### Evaluation on gene expression dataset with known grouping

We first used gene expression data of 62 bone marrow samples from leukemia patients (41 Acute Lymphocytic Leukemia (ALL), 21 Acute Myelocytic Leukemia (AML) - [42]) to test *PathME* against sNMF. The dataset is available as Bioconductor R-package golubEsets (see http://bioconductor.org/packages/release/ data/experiment/html/golubEsets.html) and has been frequently used to test clustering methods [14]. 33 out of the 41 ALL patient samples are from B-cells and 8 from T-cells. So, there are presumably *k* = 3 clusters. 1729 out of the 7129 omics features could be mapped to 211 NCI pathways.

We compared consensus sNMF applied to individual genes against *PathME* based consensus clustering in terms of the adjusted rand index [43]. According to that measure *PathME* outperformed conventional consensus sNMF clustering using individual genes as features (adj. Rand index 0.16 vs 0.02). This suggests that autoencoder based dimensionality reduction yields more statistically stable and coherent clusters, i.e. was successful in capturing relevant signal from the data.

### Multi-Omics datasets

We next applied *PathME* to four diseases with available multi-omics data from The Cancer Genome Atlas (TCGA) [44, 45] available via the cBioPortal (https:// www.cbioportal.org/) and GDC Data Portal (https://portal.gdc.cancer.go), respectively: Colorectal Cancer (CRC), Lung Squamous Cell Carcinoma (LSCC), Glioblastoma Multiforme (GBM) and Breast Cancer (PanCancer BRCA). These cancer types were chosen due to the comparable large number of patients with available multi-omics data. For each of these diseases we selected those patients where three omics data types were jointly available and pre-filtered features that were mapping to NCI cancer pathways, as previously described in Section 1.2. Details are shown in Table 1. Notably, gene expression for GBM and LSCC was based on normalized microarray data, whereas for CRC and BRCA we used RSEM estimates available from Firebrowse (http://firebrowse.org/). CNV and DNA methylation profiles were available as preprocessed microarray data.

**Table 1:** Datasets from The Cancer Genome Atlas (TCGA) used for evaluation: colorectal cancer (CRC), glioblastoma multiforme (GBM), lung squamous cell carcinoma (LSCC) and breast cancer (BRCA). Omics features correspond to those mapable to NCI pathways

Dataset	Patients	Omics types	Features
		mRNA	2295
CRC	294	miRNA	264
		CNV	2310
		mRNA	2039
GBM	273	miRNA	18
		DNA methylation	1798
		mRNA	2039
LSCC	106	miRNA	150
		DNA methylation	1846
		mRNA	2329
BRCA	747	miRNA	99
		CNV	2334

Training error curves of all autoencoder models for the optimal set of hyperparameters (found according to the procedure described in the Methods part) can be found in the Supplementary material.

We applied *PathME*, SNF and iCluster to the multi-omics data from each disease. Based on our above defined criteria for determining a good number of clusters *PathME* arrived at 5 sub-groups for CRC and BRCA, and clusters 4 for GBM and LSCC (Figure S1). Table 2 shows silhouette indices (SI) of our method compared to those obtained for SNF and iCluster. While the significantly higher SI of our method after applying consensus clustering is not unexpected, it is interesting to see that also the SI obtained from the best among 500 individual sNMA runs is clearly better compared to SNF and iCluster for GBM, BRCA and LSCC.

**Table 2:** Comparison of *PathME* vs SNF and iCluster in terms of silhouette index. For *PathME* we report the silhouette index of the consensus clustering as well as the best individual one among 500 randomly initialized sNMF runs

Cancer datasets			*PathME*		SNF	iCluster
Disease	Omics type	Cophenetic correlation	Consensus silhouette	Optimal silhouette	Silhouette	Silhouette
CRC	Number of clusters		(5)		(2)	(2)
	Multi-omics	1	0.98	0.51	0.54	0.06
GBM	Number of clusters		(4)		(2)	(3)
	Multi-omics	1	1	0.67	0.58	0.11
LSCC	Number of clusters		(4)		(4)	(2)
	Multi-omics	1	1	0.82	0.71	0.35
BRCA	Number of clusters		(5)		(2)	(3)
	Multi-omics	1	0.93	0.67	0.57	0.12

For comparison reasons we also include the results of *PathME* applied to each individual omics data type (Table 3), indicating that multi-omics based clustering via *PathME* based consensus clustering for CRC, GBM and LSCC results into at least as well discriminated patient subgroups as consensus clustering of individual omics sources. In agreement to our findings in the previous Section, *PathME* in most cases also resulted into better SI than sNMF clustering applied to individual omics features (i.e. without autoencoder based representation learning). Notably, SNF and iCluster are not applicable to individual omics types data.

**Table 3:** Comparison of *PathME* vs conventional sNMF consensus clustering of individual omics features in terms of cophenetic correlation and silhouette index

Cancer datasets		Cophenetic correlation		Consensus silhouette	
Disease	Omics type	*PathME*	sNMF (ind. features)	*PathME*	sNMF (ind. features)
CRC (5 clusters)	mRNA	1	1	0.99	0.87
	miRNA	1	1	0.97	0
	CNV	1	0.99	0.99	0.93
GBM (4 clusters)	mRNA	1	0.99	1	1
	miRNA	1	1	0.93	0.86
	Methylation	1	1	1	1
LSCC (4 clusters)	mRNA	0.92	1	0.67	1.00
	miRNA	1	1	0.98	0.98
	Methylation	1	0.99	1.00	0.97
BRCA (5 clusters)	mRNA	0.98	0.99	0.88	0.94
	miRNA	1	0.91	0.99	0.49
	CNV	1	1	1	1

### *PathME* allows for interpretable multi-Omics based patient stratification

We next analyzed clinical and biological features of the patient clusters obtained via *PathME* in the last Section.

#### Colorectal Cancer (CRC)

**Fig. 2:**
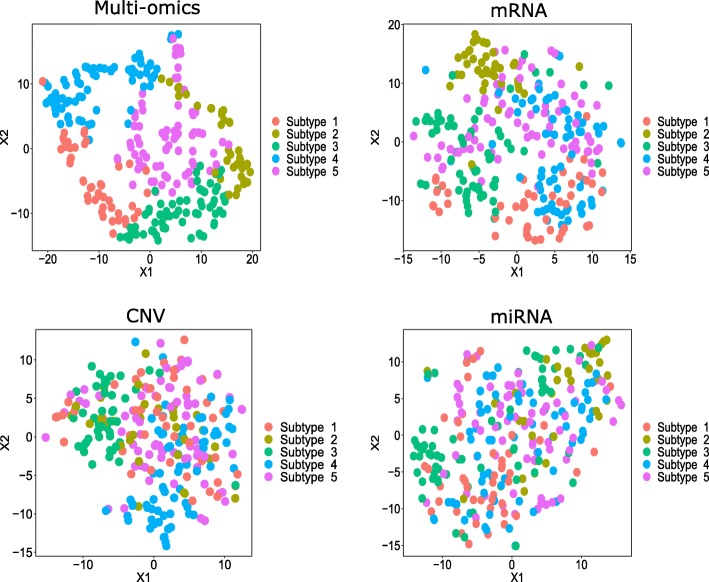
T-SNE visualization of CRC consensus clustering based on *PathME.* Data points (= patients) have been colored according to the consensus sNMF clustering of multi-omics pathway scores. T-SNE visualization of individual omics modalities is based on all features map-able to the pathways used by *PathME.* T-SNE plots for other datasets can be found in the Supplementary material

CRC patient clusters identified by *PathME* differed significantly by tissue site as well as pathological and histological stage (Table S1). Interestingly, our clusters 1-4 were highly enriched for formerly published consensus molecular subtypes (CMS) in CRC [46] (see Table S1, S2). A visual representation of the *PathME* consensus clustering via t-stochastic neighorhood embedding (t-SNE [47]) can be found in Fig. 2. We here relied on the t-SNE implementation in Bioconductor R-package M3C [48] using default parameters.

**Fig. 3:**
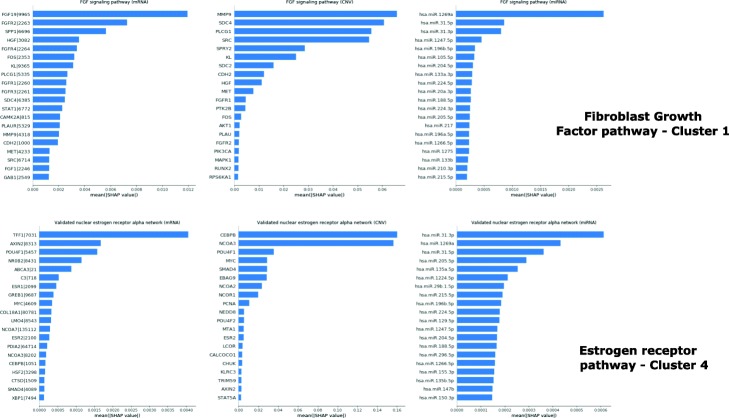
Example of SHAP analysis results obtained for CRC. For gene expression data gene symbols are shown together with Entrez gene IDs. Further results can be found in the Supplementary material, including Table S8

In the following we use examples to further demonstrate, how *PathME* can be interpreted on molecular level based on the methods described before (Fig. 3, Table S8): For cluster 1 the Fibroblast Growth Factor (FGF) signaling pathway is one of the most contributing molecular features. FGF signaling dysregulation is associated with cancer tumorgenesis and progression [49, 50]. Its components Fibroblast Growth Factor 19 (FGF19) and Fibroblast Growth Factor Receptor 2 (FGFR2) are reported to be expressed in CRC and could be useful therapeutic targets [49, 51]. Their relevance is underlined by high SHAP values. SHAP analysis also demonstrates the impact of miR-31-3p/5p. These are known to be important predictive and prognosis biomarkers in CRC [52, 53]. Altogether CNVs demonstrate the strongest impact on the multi-omics pathway score learned for FGF signaling (Table S8), and CNVs in the metalloproteinase-9 (MMP9) gene are most impactful. The MMP9 level has been suggested as a biological predictor of prognosis in CRC [54] For cluster 4, a high contribution of the nuclear estrogen receptor (ER) activation pathway is determined. ER signaling including ER-*α* and ER-*β* is implicated in CRC pathogenesis and progression and it is considered as a potential preventive and therapeutic target [55]. Estrogen Receptor 2 (ESR2), the gene encoding ER-*β*, is considered as a prognostic marker [56]. Another important pathway related to the CRC adenoma to carcinoma transition is the Ras pathway [57]. Regulation of Ras family activation is associated to cluster 5. The oncogene KRAS is considered as an important component of this pathway, because KRAS gene copy number alteration might be a useful marker to predict treatment response [58]. SHAP values demonstrate the impact of CNVs in that gene on the composite Ras signaling score, which is otherwise mostly influenced by miRNAs. The most influential miR-206 is a known prognostic factor in CRC [59].

#### Glioblastoma Multiforme (GBM)

**Fig. 4:**
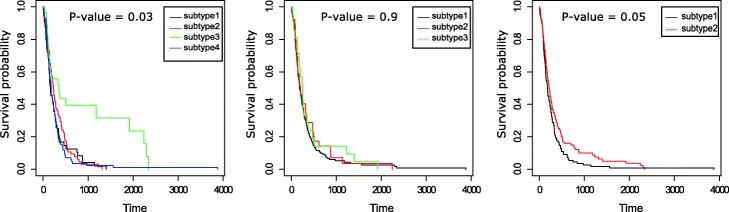
Progression free survival (days) of patients stratified by *PathME* (left), iCluster (middle) and SNF (right) for GBM. *P*-values were corrected for the confounding effect of age and adjusted for multiple testing

A t-SNE visualization of the *PathME* consensus clustering can be found in Figure S4. GBM patient clusters found by *PathME* differed significantly by PFS (*FDR* = 0*.*03; iCluster: *FDR* = 0*.*9; SNF: *FDR* = 0.05 – Fig. 4), gender distribution (in agreement with recent observations that gender may impact cancer survival.

[60]), Verhaak’s molecular classification [2] and MGMT methylation status [61] (Tables S3, S4). *PathME* cluster 3 (green PFS curve) showed the best prognosis of patients and an enrichment of somatic mutations in Isocitrate Dehydrogenase 1 (IDH1), which is a well-known positive prognostic factor in GBM [62]. Notably, this enrichment of somatic mutations was not found in clustering results of SNF and iCluster. Cluster 3 also showed a strong enrichment of the proneural Verhaak subtype, which has been associated to positive prognosis. In contrast, cluster 4 (blue PFS curve) revealed a high enrichment of the mesenchymal subtype that has been associated with poor prognosis [2].

By investigating the sNMF based matrix factorization we can find that cluster 4 is specifically associated to transcriptional targets of c-MYC, which has been associated with disease prognosis [63]. Interestingly, there is also a significant difference in the fraction of mutated genes in that NCI pathway across clusters (*FDR* = 0*.*03, Kruskal-Wallis test, Figure S7).

Cluster 2 is associated to Platelet-Derived Growth Factor Receptor-*β* (PDGFR-*β*) signaling and Transforming Growth Factor -*β* (TGF-*β*) receptor signaling. PDGFR and TGF-*β* pathways are known to be dysregulated in GBM and they contribute to its pathogenesis and progression [64]. PDGFR and TGF-*β* are are currently considered as therapeutic targets [64, 65]. In addition, TGF-*β* activity is associated with differences in prognosis in gliomas, including GBM [65, 66]. Investigation of SHAP values allows for gaining additional insight (Table S9): The most contributing gene to the PDGFR signaling pathway score is transgelin-2, which has recently been found to be expressed at significantly higher levels in IDH1 WT patients. The most influential data modalities on the multi-omics score for TGF-*β* signaling are miRNAs and DNA methylation, and cg15001381 is the most contributing CpG. This CpG is in the promotor region of AXIN1, a gene that has been reported to be downregulated in GBM [67]. More results can be found in the Supplementary material.

#### Lung squamous cell carcinoma (LSCC)

LSCC patient clusters identified by *PathME* showed significant differences in lymph node pathology, race, tissue source site and tumor size (Table S5). A visual representation of the *PathME* consensus clustering via t-SNE can be found Figure S5.

According to the analysis of the *PathME* model (Table S10), cluster 1 is associated to *α*6*β*4 integrin-ligand interactions. The *α*6*β*4 signaling pathway is known to play a role in many cellular processes in human malignancies including LSCC. its alteration induces aggressive behavior [68]. In LSCC, *α*6*/β*4 integrin encoded by Integrin Alpha-6 (ITGA6) and Integrin Beta-4 (ITGB4) genes (high SHAP values), respectively, have been found moderately expressed and *β*4 integrin was highly expressed [69]. MiRNAs are the most influential data modality for the multi-omics score of that pathway according to SHAP, and miR-149 is the most impactful miRNA. It has been associated with the Epithelial-to-Mesenchymal Transition (EMT) phenotype in Non-Small-Cell Lung Cancer (NSLCC) [70]. Moreover, SHAP values show that the Murine Double Minute 2 (MDM2) gene has a high impact on the androgen receptor activity pathway, which was found as a feature for cluster 4. MDM2 was found to strongly correlate with patient survival in NSCLC [71]. For miR-21, associated to the Ataxia Telangiectasia Mutated (ATM) pathway (cluster 4), and miR-155, associated to calcineurin-dependent Nuclear Factor of Activated T-cells (NFAT) signaling in lymphocytes (cluster 2), expression has been described as a prognosis factor in NLSCC, including LSCC [72, 73].

#### Breast Cancer (BRCA)

A visual representation of the *PathME* consensus clustering via t-SNE can be found Figure S6. BRCA patient clusters found by *PathME* showed significant differences in race and histology type (Table S6). Moreover, there was a strong correlation with the common molecular classification into luminal A, luminal B, normal-like, basal-like and HER2 enriched subtypes [74] (Table S7). Cluster 1 and 2 were highly enriched for the luminal B subtype, cluster 3 for the basal-like subtype, cluster 4 for the liminal A subtype and cluster 5 for the normal-like subtype. Cluster 3 showed a strong enrichment of TP53 somatic mutations (*p <* 1*E* − 11), and cluster 4 of somatic mutations in Cadherin-1 (CDH1), GATA3 and Phosphatidylinositol4,5-bisphosphate 3-Kinase Catalytic subunit Alpha (PIK3CA). All these are known prognostic factors for BRCA [75, 76].

According to the further inspection of the *PathME* model (Table S11), cluster 1 is associated to the stabilization and expansion of the E-cadherin adherens junction pathway, which has been associated with tumor survival in several cancers including breast [77]. There is a significant difference in the fraction of genes carrying somatic mutations in this pathway across clusters (Figure S9). According to SHAP analysis data the most influential gene in the pathway is Insulin-like Growth Factor 1 Receptor (IG1FR), which has an important role in tumor growth and survival [78]. MiR-184 is the most impactful miRNA in the same pathway. It has been discussed as a predictive biomarker for breast cancer [79]. Cluster 4 is highly associated to CDC42 (Cell Division Control) signaling. CDC42 has been discussed as a drug target in breast cancer [80]. According to SHAP a CNV in the Ribosomal Protein S6 Kinase B1 (RPS6KB1) has a high impact on the pathway score. Indeed copy number alterations in this gene have been associated to prognosis of breast cancer patients [81].

Once again, these are only examples and more results can be found in the Supplements to this paper. Altogether they indicate the ability to interpret *PathME* clusters via their association to clinical and molecular features.

## Discussion

We could show that *PathME* is better able to recover known patient leukemia sub-groups than consensus sparse NMF clustering using individual omics features. Additional analysis of 12 individual omics datasets (3 per disease – Table 3) with unknown subgroups in most cases resulted into a higher silhouette index for *PathME* compared to sNMF.

For multi-omics data we observed higher silhouette indices compared to SNF and iCluster. In contrast to these methods *PathME* is not limited to multi-omics data, but can also be applied to a single data modality. A further advantage of *PathME* is that it can effectively address the high dimensionality of omics data due to the proposed multi-modal autoencoder architecture. At the same time this approach yields interpretable results via investigation of sNMF results and SHAP analysis, as demonstrated above.

Of course, *PathME* is not without limitations: Instead of auto-encoding each pathway separately one could try to take advantage of the fact that pathways are not independent from each other and design the neural network model such that all pathway scores are jointly learned. However, this approach – although theoretically being more elegant – would not only yield a drastic increase of computation time, but also of the number of parameters to be learned, hence raising the risk of overfitting due to the high dimensionality of omics data. It is worthwhile to emphasize that the proposed modular architecture of our multi-modal autoencoder heavily reduces the number of trainable weights compared to a fully connected standard autoencoder architecture. Our approach is therefore in essence a compromise between a realistic consideration of sample sizes in omics data and affordable model complexity in the light of the typical high dimensionality of omics data. In that context we should also mention that we use multiple regularization techniques (drop-out, weight penalties) to further address this point.

## Conclusion

The overall contribution of this work is two-fold:

1. We suggested a multi-modal sparse denoising autoencoder architecture that allows for an effective and interpretable combination of multi-omics data and pathway information. Our specific model addresses the high dimensionality of omics data.
2. Our second contribution is to demonstrate that patient specific pathway scores derived from our autoencoder model allow for a robust and interpretable disease subtype identification. Disease sub-groups are tighter (i.e. have higher silhouette indices) than the ones produced by SNF and iCluster. Our results based on different cancer datasets demonstrate the association of patient subgroups identified by our method to relevant clinical and biological features.

Altogether we see our proposed model as a step towards a more effective integration of multi-omics data in the context of precision medicine by using modern data science techniques. As a next step we could use pathway profiles in a supervised learning context by changing the loss function of our multi-modal neural network accordingly.

## Supplementary information

**Additional file 1.** Supplementary text, including links to further online material.

**Additional file 2. ***PathME* code (python) and additional analysis codes: https://github.com/AminaLEM/PathME.

## Data Availability

All data used in this work are publicly available via https://www.cbioportal.com, https://www.portal.gdc.cancer.go, http://firebrowse.org and https://www.bioconductor.org/packages/release/data/experiment/html/golubEsets.html. *PathME* code is available under https://github.com/AminaLEM/PathME.

## Abbreviations

PathME: PATHway based Multi-modal sparse autoEncoders sNMF: sparse Non-negative Matrix Factorization
SNF: Similarity Network Fusion
SHAP: Shapley additive exPlanations
SI: Silhouette Index
TCGA: The Cancer Genome Atlas
GDC: Genomic Data Commons
GBM: Glioblastoma Multiforme
CRC: Colorectal Cancer
LSCC: Lung Squamous Cell Carcinoma
BRCA: Breast Cancer
NSLCC: Non-Small-Cell Lung Cancer
ALL: Acute Lymphocytic Leukemia
AML: Acute Myeloacytic Leukemia
CNV: Copy Number Variation
GISTC2: Genomic Identification of Significant Targets in Cancer
FDR: False Discovery Rate
OS: Overall Survival
PFS: Progression Free Survival
DFS: Disease Free Survival
Nadam: Nesterov-accelerated Adam
ATM: Ataxia-Telangiectasia Mutated
CDC42: Cell Division Control 42
CDH1: Cadherin 1
EMT: Epithelial-tomesenchymal Transition
ER: Estrogen Receptor
ESR2: Estrogen Receptor 2
FGF: Fibroblast Growth Factor
FGF19: Fibroblast Growth Factor 19
FGFR2: Fibroblast Growth Factor Receptor 2
IDH1: Isocitrate Dehydrogenase 1
IG1FR: Insulin-like Growth Factor 1 Receptor
ITGA6: Integrin alpha-6
ITGB4: Integrin beta-4
MDM2: Murine Double Minute 2
NFAT: Nuclear Factor of Activated T-cells
PDGFR: Platelet-Derived Growth Factor Receptor
PIK3CA: Phosphatidylinositol-4,5-bisphosphate 3-Kinase Catalytic subunit Alpha
RPS6KB1: Ribosomal Protein S6 Kinase B1 TGF-*β*: Transforming Growth Factor-*β*
