# Supplementary material for: PathME: pathway based multi-modal sparse autoencoders for clustering of patient-level multi-omics data
Source: BMC Bioinformatics. 2020 Apr 16;21:146. doi: 10.1186/s12859-020-3465-2 (PMC7161108; doi:10.1186/s12859-020-3465-2)
Supplement: Supplementary file 1 — Additional file 1. Supplementary text, including links to further online material. [file 12859_2020_3465_MOESM1_ESM.pdf]

PathME: Pathway based Multi-modal Sparse Autoencoders for Unsupervised Clustering of Patient-Level Multi-Omics Data

Amina Lemsara, Salima Ouadfel, Holger Fröhlich

1. Estimation of Optimal Cluster Number

CRC

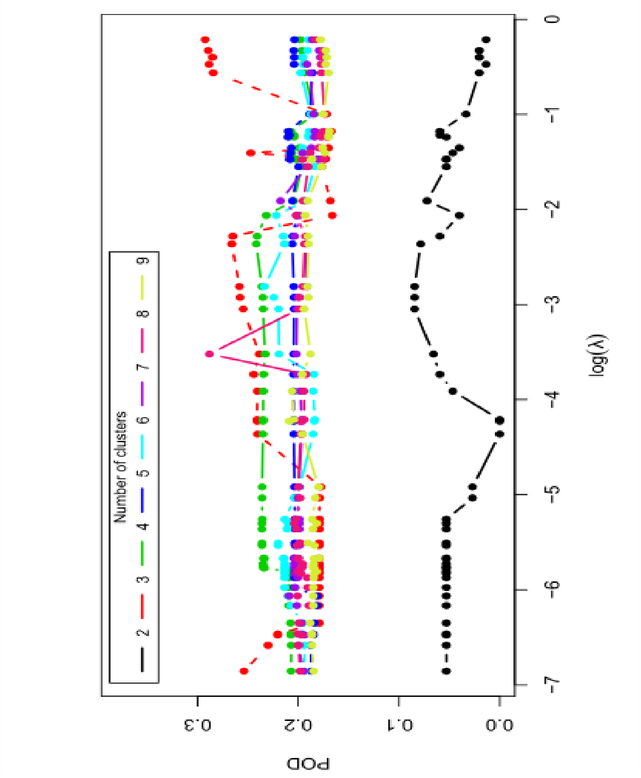

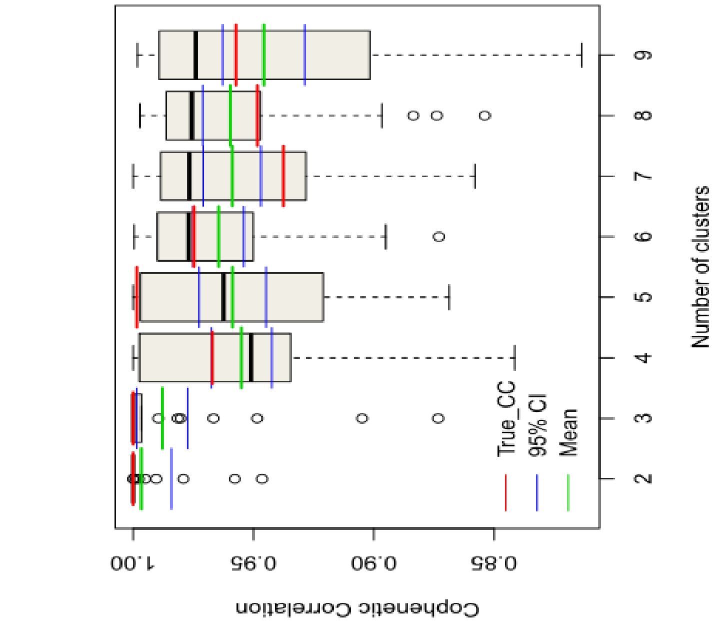

GBM

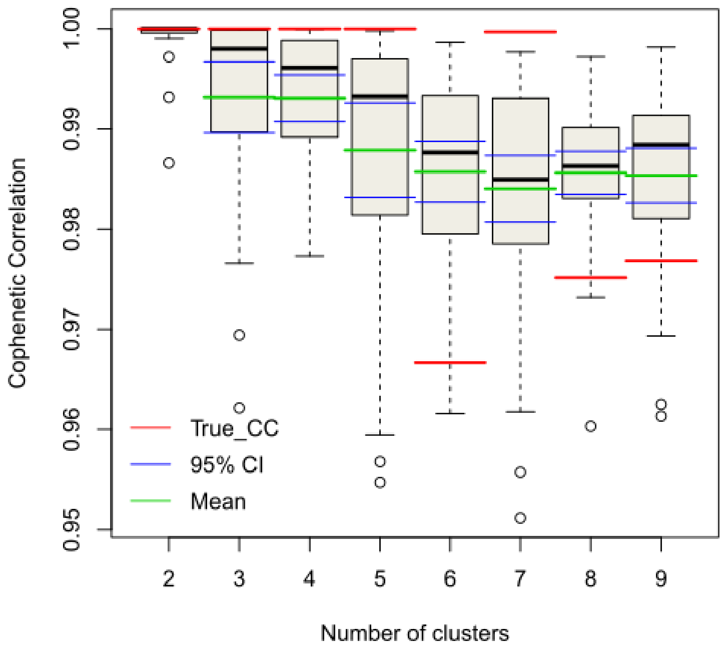

Cophenetic Correlation
1.00
0.99
0.98
0.97
0.96
0.95
True_CC
95% CI
Mean
2
3
4
5
6
7
8
9
Number of clusters

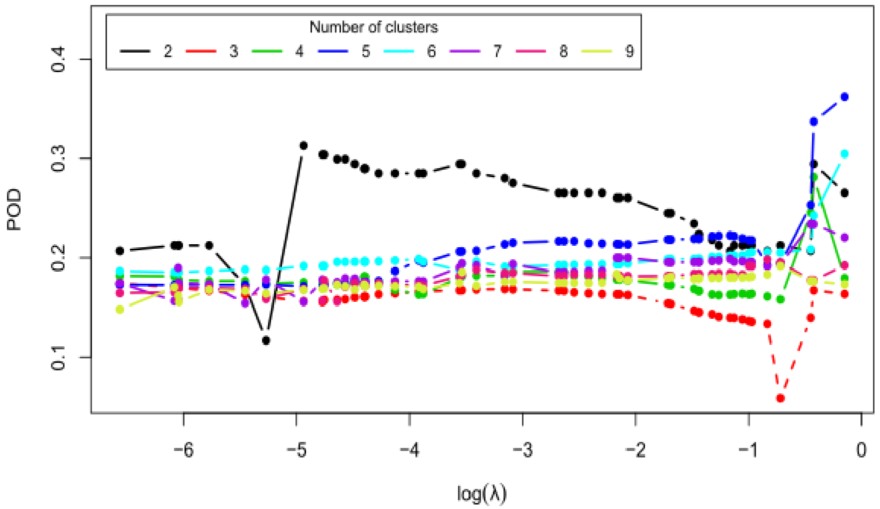

Number of clusters
2
3
4
5
6
7
8
9
POD
0.4
0.3
0.2
0.1
-6
-5
-4
-3
-2
-1
0
log(λ)

LSCC

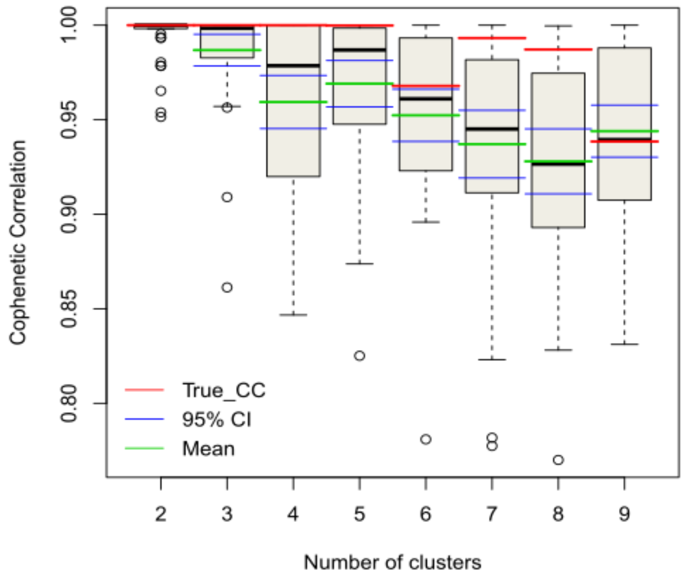
Cophenetic Correlation
1.00
0.95
0.90
0.85
0.80
True_CC
95% CI
Mean
2
3
4
5
6
7
8
9
Number of clusters

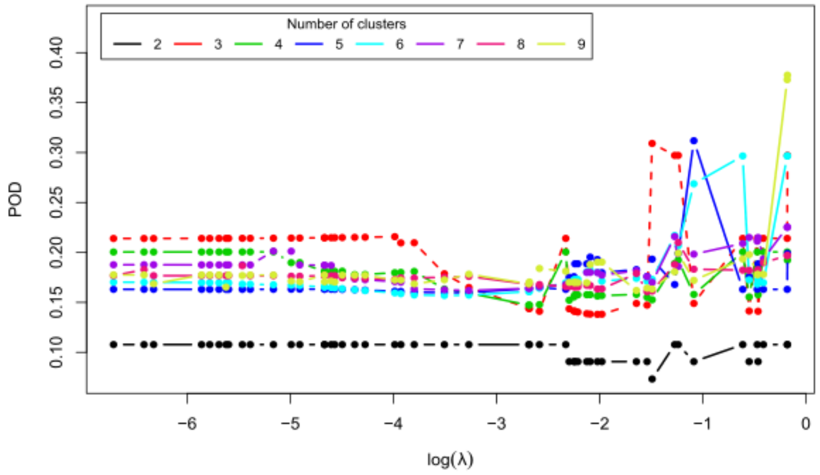
Number of clusters
2
3
4
5
6
7
8
9
POD
0.40
0.35
0.30
0.25
0.20
0.15
0.10
−6
−5
−4
−3
−2
−1
0
log(λ)

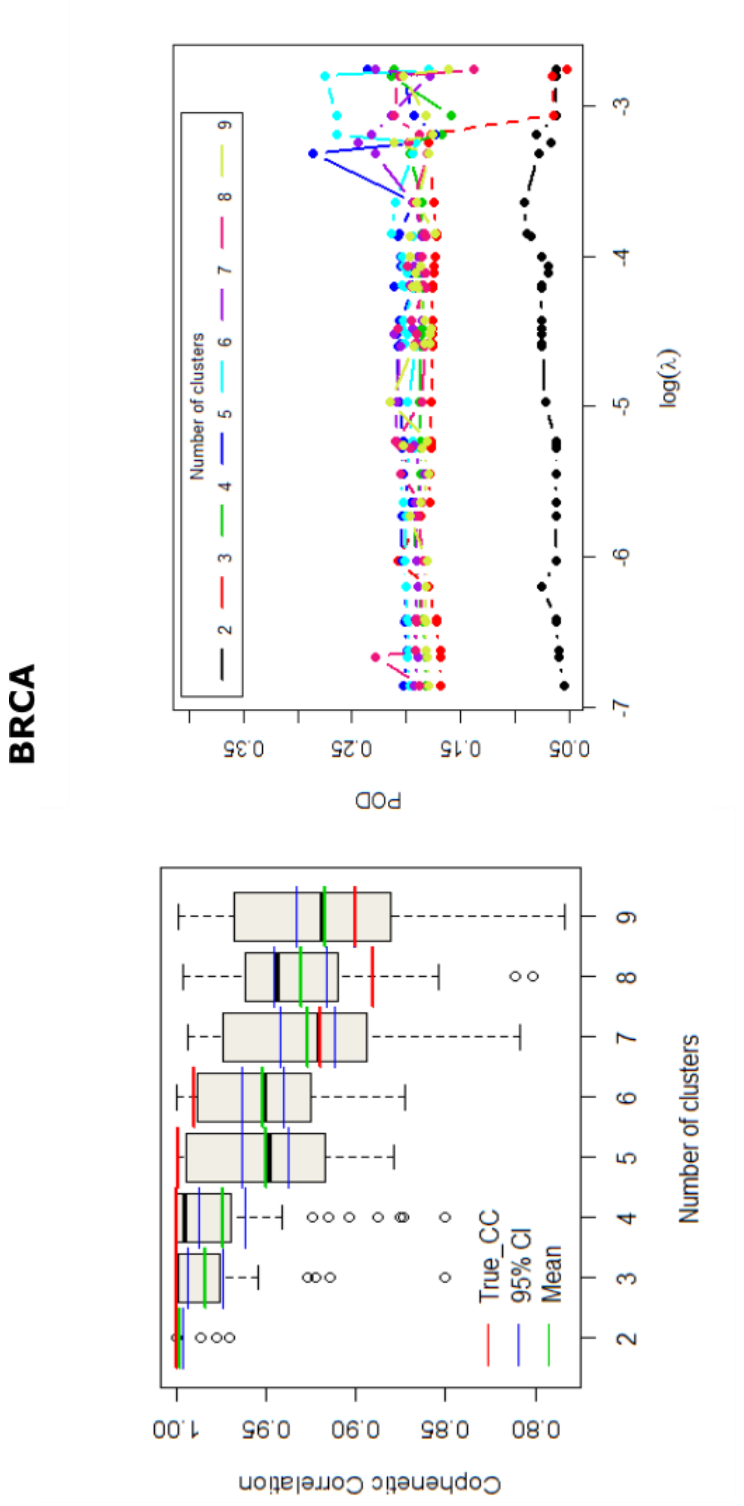

Fig S1. Determination of number of clusters for CRC, GBM, LSCC and BRCA: Left) Comparison of true cophenetic correlation compared to the distribution of the cophenetic correlation resulting from 40 random permutations of the data. Right) Proportion of Deviance (POD) score for iCluster method. The plot shows 50 values of □ chosen uniformly from [0.001, 0.9] for 2, 3, …, 9 number of clusters.

2. Association of Clusters with Clinical Features

Significances of categorical variables were tested via a $\chi 2$ test, including the agreement with existing molecular classification schemes. For numerical variables a one-way ANOVA was applied. For survival we first checked nominal significance of the association to age via a Cox regression model that only contained age as predictor. If this was true, we fitted a Cox regression model that contained a factor "cluster" plus age as predictors. This model was then compared against the "null" Cox regression model that only contained age as predictor. Both models were compared via a likelihood ratio test, and the corresponding p-value was reported as the significance of the age corrected association to the clustering. In case of no significant association with age a conventional log-rank test was used.

To test the association with MGMT methylation status in GBM we applied a MANOVA and a global test (Goeman et al., 2004).

Multiple testing correction of p-values was performed via Benjamini & Hochberg's method [1] to control false discovery rate (FDR) separately for each method (i.e. column).

Table S1: FDR for association of clusters with clinical features (CRC).

Clinical features	*PathME* (K=5)				SNF (K= 2)	iCluster (K=2)
	Multi-omics	mRNA	CNV	miRNA		
Tumor tissue site	1.58E-02	6.83E-01	1.73E-02	1.89E-01	4.27E-06	8.34E-03
Pathologic stage	1.15E-02	4.66E-01	4.52E-02	9.68E-01	1.86E-02	8.84E-01
Pathology T_stage	1.28E-01	4.66E-01	6.00E-01	7.51E-01	3.92E-01	8.84E-01
Pathology N_stage	6.78E-02	3.99E-01	3.32E-01	1.21E-01	3.63E-02	7.20E-01
Pathology M_stage	1.24E-01	6.83E-01	6.65E-01	7.51E-01	3.84E-02	8.84E-01
Gender	5.71E-01	6.20E-01	9.70E-01	7.51E-01	6.05E-01	8.84E-01
Radiation therapy	8.30E-02	9.25E-01	2.39E-01	7.26E-01	6.05E-01	8.84E-01
Histological type	1.49E-03	6.20E-01	1.90E-03	8.70E-02	2.05E-09	4.72E-05
Age	7.25E-01	6.60E-01	5.21E-01	5.75E-02	5.44E-01	8.94E-01
Overall Survival (OS)	8.30E-02	6.00E-02	6.00E-01	8.70E-02	1.03E-01	7.20E-01

Progression free survival (PFS)	2.67E-01	6.00E-02	6.00E-01	8.70E-02	1.03E-01	8.84E-01
Disease free survival (DFS)	9.00E-01	6.20E-01	9.00E-02	1.89E-01	1.50E-01	7.20E-01
Enrichment of known prognostic somatic mutations (KRAS, NRAS, BRAF, PTEN)	No somatic mutations of these prognostic markers were found in our data					

Table S2: Enrichment analysis of known CRC subtypes.

Method		Subtypes	Consensus Molecular Subtypes (CMSs)			
			CMS1	CMS2	CMS3	CMS4
PathME	Multi-omics	Subtype 1		6.26E-03	7.03E-05	
		Subtype 2				7.88E-16
		Subtype 3	5.10E-19		1.61E-05	
		Subtype 4		1.70E-10		
		Subtype 5				
	mRNA	Subtype 1		1.94E-13		
		Subtype 2	4.36E-09		1.19E-05	
		Subtype 3				5.45E-03
		Subtype 4				3.03E-13
		Subtype 5				3.33E-04
	miRNA	Subtype 1		1.06E-05		
		Subtype 2				4.11E-06
		Subtype 3				9.21E-06
		Subtype 4				
		Subtype 5				
	CNV	Subtype 1				

		Subtype 2		1.81E-03		
		Subtype 3		1.97E-06		
		Subtype 4				2.49E-02
		Subtype 5	3.17E-11		3.17E-06	
SNF		Subtype 1	2.93E-21		1.04E-05	
		Subtype 2				
iCluster		Subtype 1	2.18E-10		5.91E-09	
		Subtype 2		1.21E-12		

Table S3: FDR for association of clusters with clinical features (GBM).

		PathME (K=4)				SNF (K=2)	iCluster (K=3)
		Multi-omics	mRNA	DNA methylation	miRNA		
MGMT	(Manova, Pillai test)	6.44E-10	1.20E-03	2.55E-11	4.52E-02	3.39E-05	5.51E-01
	(Global test)	6.72E-08	2.80E-04	2.04E-08	2.87E-03	6.48E-08	8.34E-01
Gender		2.77E-03	3.61E-02	1.04E-02	2.66E-01	4.57E-01	1.95E-01
History_of_neoadjuvant_treatment		3.87E-01	8.96E-06	1.19E-01	9.31E-02	4.61E-01	1.14E-09
Age		1.19E-08	1.03E-02	2.04E-08	5.17E-01	3.96E-01	1.71E-02
OS, corrected for age		3.87E-01	1.65E-01	3.47E-01	2.66E-01	4.80E-02	5.51E-01
PFS, corrected for age		3.31E-02	5.02E-01	1.87E-02	1.33E-02	4.80E-02	8.98E-01
Enrichment of known prognostic somatic mutations		Subtype 3 IDH1: 2.55E-06		Subtype 2 IDH1: 1.76E-06			
Verhaak classification		6.59E-31	8.48E-14	3.20E-24	5.65E-14	1.31E-15	6.68E-08

Table S4: Enrichment analysis of GBM subtypes for Verhaak subtypes.

Method		Subtypes	Verhaak classes			
			Classical	Mesenchymal	Neural	Proneural
PathME	Multi-omics	Subtype 1				7.92E-09
		Subtype 2	1.76E-11			
		Subtype 3				8.69E-10
		Subtype 4		3.21E-10	7.64E-03	
	mRNA	Subtype 1			1.28E-04	
		Subtype 2		1.51E-08		
		Subtype 3				
		Subtype 4	3.09E-02			1.63E-03
	miRNA	Subtype 1				2.58E-08
		Subtype 2		2.26E-08		
		Subtype 3			1.21E-02	
		Subtype 4	7.29E-03			
	Methylation	Subtype 1				9.10E-05
		Subtype 2				2.79E-10
		Subtype 3		4.42E-08		
		Subtype 4	6.83E-08			
SNF		Subtype 1		4.12E-09	2.40E-04	
		Subtype 2	4.85E-02			1.31E-11
iCluster		Subtype 1				
		Subtype 2		2.56E-05		
		Subtype 3		3.23E-02		

Table S5:FDR for association of clusters with clinical features (LSCC).

	PathME (K=4)				SNF (K=4)	iCluster (K=2)
	Multi-omics	mRNA	DNA methylation	miRNA		
Ajcc_metastasis_pathologic pm	5.53E-01	7.23E-01	4.94E-01	1.00E+00	6.90E-01	9.62E-01
Ajcc_nodes_pathologic_pn	9.38E-03	7.95E-01	4.94E-01	1.00E+00	6.53E-01	9.62E-01
Ajcc_pathologic_tumor_stage	8.86E-02	7.23E-01	4.94E-01	1.00E+00	6.53E-01	9.62E-01
Ajcc_tumor_pathologic_pt	5.53E-01	2.13E-01	9.18E-01	1.58E-01	6.53E-01	9.62E-01
History_neoadjuvant_trtyn	2.75E-01	7.14E-01	4.94E-01	1.00E+00	6.53E-01	1.00E+00
History_other_malignancy	5.53E-01	7.95E-01	8.53E-01	3.71E-01	8.34E-01	9.62E-01
Primary_site_patient	5.53E-01	7.95E-01	5.44E-01	1.00E+00	8.34E-01	9.62E-01
Race	2.76E-03	7.14E-01	2.49E-04	1.00E+00	7.17E-03	4.98E-02
Residual_tumor	5.53E-01	6.00E-01	4.94E-01	1.00E+00	6.53E-01	9.62E-01
Gender	2.20E-01	7.95E-01	4.94E-01	1.00E+00	7.64E-01	9.62E-01
Tissue_source_site	8.93E-14	6.36E-02	1.59E-10	1.31E-11	1.65E-12	1.22E-11
Tobacco_smoking_history indicator	9.30E-02	7.23E-01	5.44E-01	1.00E+00	6.53E-01	8.98E-01
Age	5.53E-01	7.95E-01	5.44E-01	7.20E-01	9.48E-01	9.62E-01
Fraction_genome_altered	1.05E-01	6.01E-03	4.94E-01	7.59E-03	9.69E-01	4.83E-01
Longest_dimension	4.09E-02	1.22E-01	4.94E-01	8.14E-01	8.33E-03	9.62E-01
Shortest_dimension	4.09E-02	8.44E-01	7.35E-02	1.00E+00	9.48E-01	1.98E-02
Specimen_second_longest dimension	6.72E-01	7.32E-01	6.49E-01	8.35E-01	6.53E-01	9.62E-01

Smoking_pack_years	5.53E-01	7.95E-01	4.94E-01	1.00E+00	8.96E-01	4.20E-01
Overall survival (OS)	7.00E-01	6.00E-01	4.94E-01	3.71E-01	6.53E-01	8.98E-01
Progression free survival (PFS)	5.53E-01	6.00E-01	4.94E-01	1.58E-01	5.25E-01	4.20E-01
Disease free survival (DFS)	5.53E-01	7.95E-01	8.53E-01	1.00E+00	6.53E-01	9.62E-01
Enrichment of known prognostic somatic mutations (KRAS, EGFR, P53)	Not significant					

Table S6: FDR for association of clusters with clinical features (BRCA).

	PathME (K=5)				SNF (K=2)	iCluster (K=3)
	Multi-omics	mRNA	CNV	miRNA		
Gender	2.19E-08	6.73E-02	1.05E-04	9.90E-01	4.46E-01	2.05E-02
Race	2.19E-08	4.29E-02	4.98E-02	1.56E-04	3.55E-09	6.93E-02
ajcc_pathologic_tumor_stage	1.33E-01	2.85E-01	5.61E-01	5.48E-01	6.00E-02	3.72E-01
histological_type	2.19E-08	8.46E-09	7.96E-04	4.32E-02	1.21E-15	2.35E-08
age_at_initial_pathologic_diagnosis	8.36E-02	4.29E-02	2.20E-01	2.89E-01	1.88E-01	1.78E-01
Overall survival (OS) after age correction	1.33E-01	4.29E-02	3.67E-01	6.04E-01	6.60E-01	1.10E-01
Disease-specific survival (DSS) after age correction	1.39E-01	1.07E-01	3.67E-01	1.00E+00	6.60E-01	3.72E-01
Disease-free interval (DFI) after age correction	3.30E-01	4.40E-01	3.67E-01	5.50E-01	3.67E-01	2.20E-02
Progression free interval (PFI) after age correction	2.93E-01	2.44E-01	8.00E-01	9.90E-01	6.60E-01	9.00E-01
Progression free survival (PFS) after age correction	3.08E-01	7.40E-02	3.67E-01	1.83E-01	1.00E+00	8.80E-01

Enrichment of known prognostic somatic mutations (TP53, CDH1,GATA3, PIK3CA)	**Subtype 3:** TP53: 1.04E-12 **Subtype 4:** CDH1: 0.898E-3 GATA3: 0.18E-2 PIK3CA: 1.28E-02	-	-	**Subtype 1:** ZFYVE26: 2.70E-02	-	**Subtype 1:** TP53: 3.80E-03
Molecular classification	2.42E-15	7.54E-08	2.42E-15	6.04E-01	1.21E-15	2.42E-15

Table S7: Enrichment analysis of known BRCA subtypes.

Method		Subtypes	Molecular Subtypes				
			Luminal A	Luminal B	basal-like	HER2-enriched	Normal-like
PathME	Multi-omics	Subtype 1		6.11E-05			
		Subtype 2		9.79E-14			
		Subtype 3			3.67E-34		
		Subtype 4	6.38E-16				
		Subtype 5					4.17E-09
	mRNA	Subtype 1					
		Subtype 2			3.00E-02		
		Subtype 3			7.30E-04		1.00E-02
		Subtype 4					
		Subtype 5	3.24E-03				
	miRNA	Subtype 1	Not significant				
		Subtype 2					
		Subtype 3					
		Subtype 4					
		Subtype 5					
	CNV	Subtype 1		1.46E-04			

		Subtype 2		8.75E-08			
		Subtype 3		7.43E-03		6.16E-07	
		Subtype 4	3.18E-06				
		Subtype 5					8.64 3E-09
SNF		Subtype 1			4.86E-80		
		Subtype 2					
iCluster		Subtype 1		3.13E-05	3.91E-06		
		Subtype 2	2.04E-14				1.49E-03
		Subtype 3		2.48E-05			

3. Basis and Coefficient maps for Multi-Omics Clustering

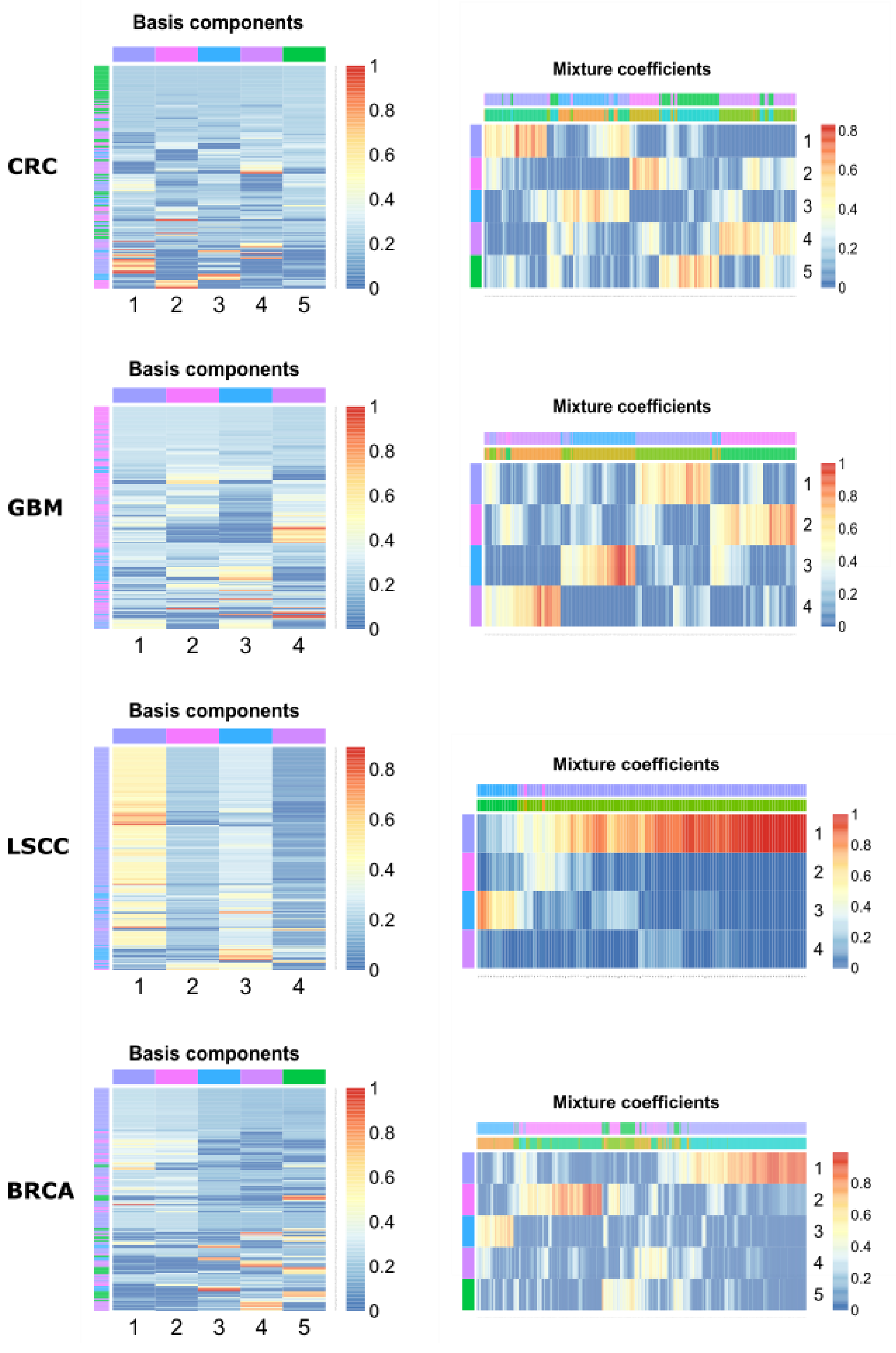

Fig S2: Basis and coefficient maps for the optimal solution among 500 individual sNMF runs for *PathME*.

4. Visualization of Clustering

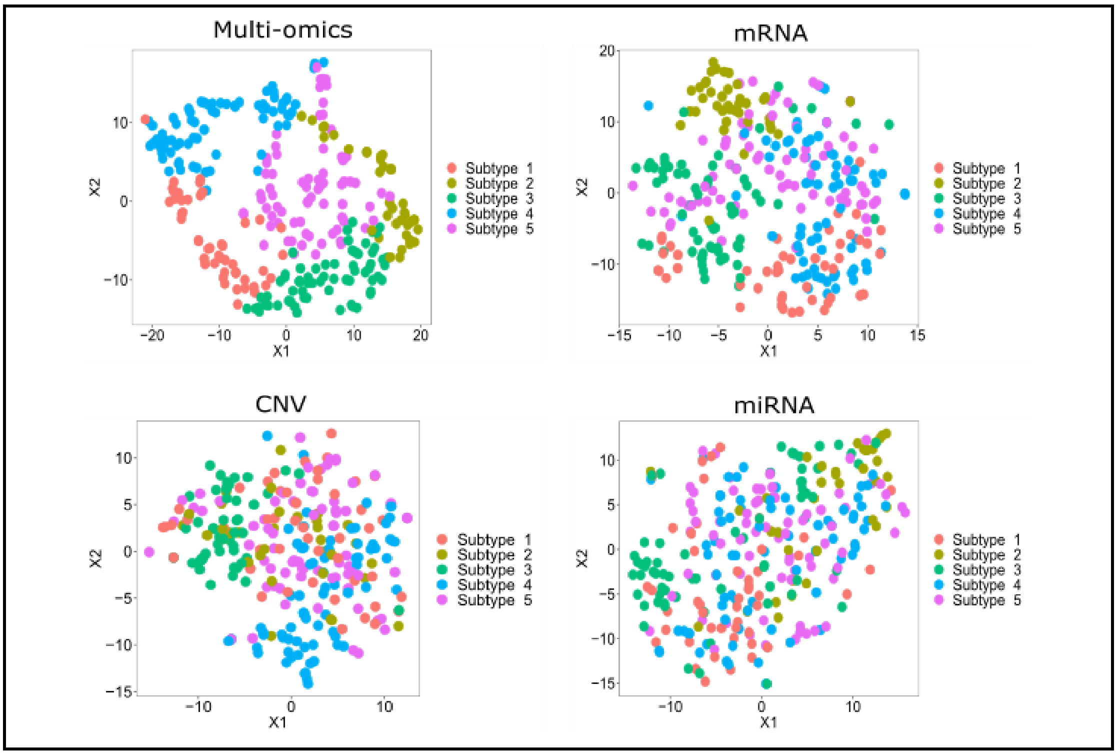

Fig S3: T-SNE visualization of CRC consensus clustering based on *PathME*. Data points have been colored according to the consensus sNMF clustering of multi-omics pathway scores. T-SNE visualization of individual omics modalities is based on all features map-able to the pathways used by *PathME*.

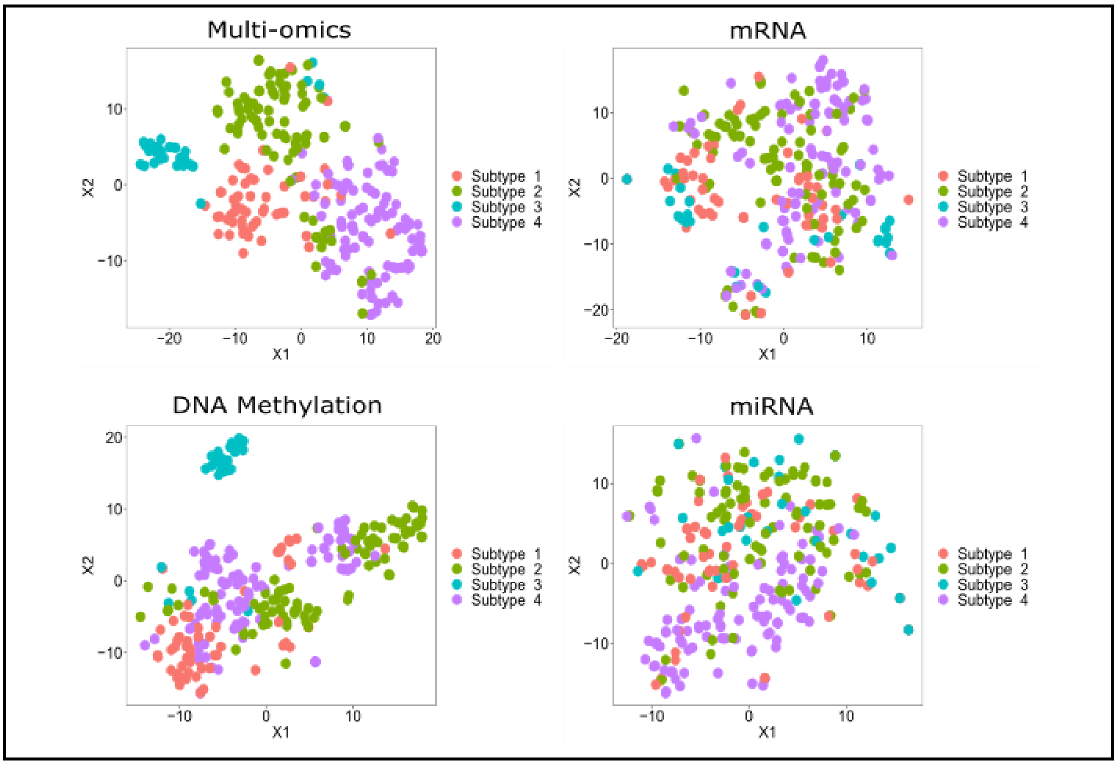

Fig S4: T-SNE visualization of GBM consensus clustering based on *PathME*. Data points have been colored according to the consensus sNMF clustering of multi-omics pathway scores. T-SNE visualization of individual omics modalities is based on all features map-able to the pathways used by *PathME*.

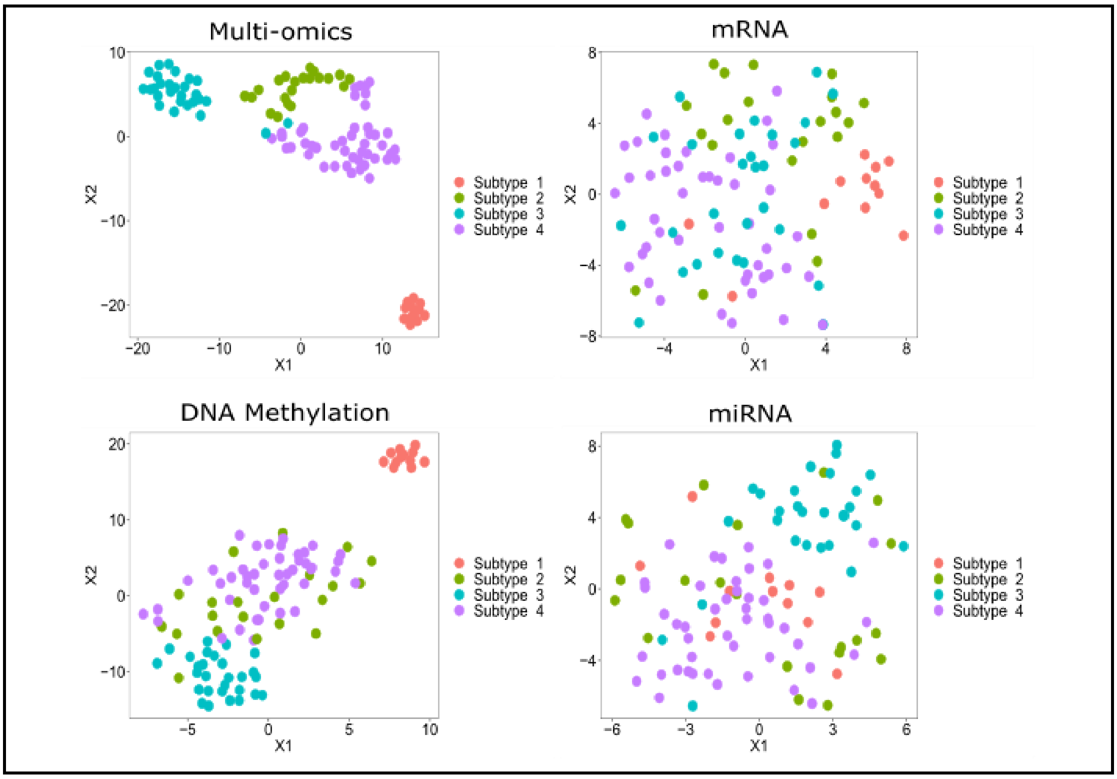

Fig S5: T-SNE visualization of LSCC consensus clustering based on *PathME*. Data points have been colored according to the consensus sNMF clustering of multi-omics pathway scores. T-SNE visualization of individual omics modalities is based on all features map-able to the pathways used by *PathME*.

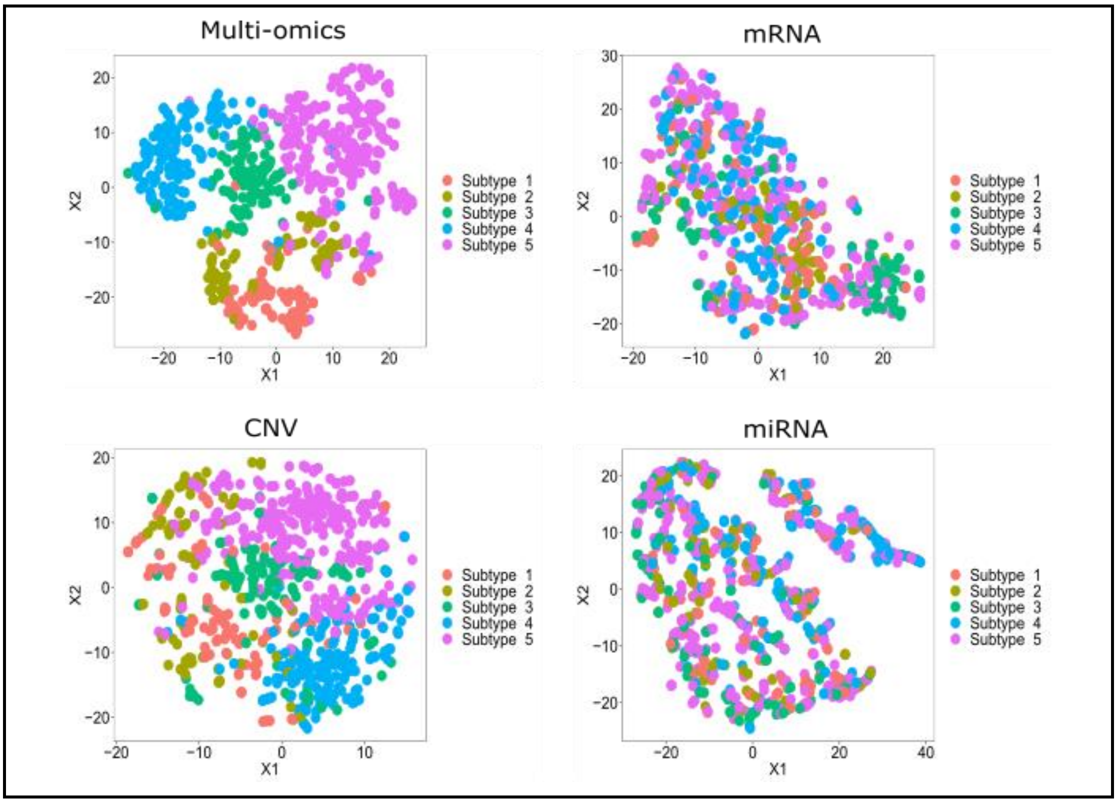

Fig S6: T-SNE visualization of BRCA consensus clustering based on *PathME*. Data points have been colored according to the consensus sNMF clustering of multi-omics pathway scores. T-SNE visualization of individual omics modalities is based on all features map-able to the pathways used by *PathME*.

5. Cluster specific pathways and impact of individual omics types

Table S8: Top 2 pathways selected for each CRC subtype and mean absolute SHAP values per pathway and omics type.

Subtype 1		Subtype 4	
FGF signaling pathway	mRNA: 0.0154<br>CNV: 0.076<br>miRNA:7.8E-05	Validated nuclear estrogen receptor alpha network	mRNA: 0.0004<br>CNV: 0.01<br>miRNA:1.2E-04
Syndecan-4-mediated signaling events	mRNA : 0<br>CNV: 0.01<br>miRNA:0.0008	Validated nuclear estrogen receptor beta network	mRNA : 0.0004<br>CNV: 0.02<br>miRNA:0.0004
**Subtype 2**		**Subtype 5**	
Syndecan-1-mediated signaling events	mRNA : 0.0018<br>CNV: 0.008<br>miRNA:0.0014	Regulation of Ras family activation	mRNA: 0<br>CNV: 3.1E-10<br>miRNA:4.78E-10

ATF-2 transcription factor network	mRNA : 0.0004<br>CNV: 0.0006<br>miRNA:2.4E-05	amb2 Integrin signaling	mRNA: 0.0002<br>CNV: 0.0008<br>miRNA:0.0008
Subtype 3			
Alpha9 beta1 integrin signaling events	mRNA: 0<br>CNV: 1.7E-06<br>miRNA:1.04E-08		
Beta1 integrin cell surface interactions	mRNA: 1.72E-04<br>CNV: 6.9E-05<br>miRNA: 1.2E-05		

Table S9: Top 2 pathways selected for each GBM subtype and mean absolute SHAP values per pathway and omics type.

Subtype 1		Subtype 3	
IL23-mediated signaling events	mRNA: 5.9E-05<br>DNA methylation: 0.0002<br>miRNA: 3.75E-05	BCR signaling pathway	mRNA: 1.4E-05<br>DNA methylation: 1.9E-04<br>miRNA: 1.25E-08
Urokinase-type plasminogen activator (uPA) and uPAR-mediated signaling	mRNA: 5.71E-11<br>DNA methylation: 1.94E-08<br>miRNA: 4.6E-12	Regulation of Ras family activation	mRNA: 0.007<br>DNA methylation: 0.027<br>miRNA: 0.008
Subtype 2		Subtype 4	
PDGFR-beta signaling pathway	mRNA: 0.001<br>DNA methylation: 0.016<br>miRNA: 0	Signaling events mediated by HDAC Class I	mRNA: 9.09E-05<br>DNA methylation:0.01<br>miRNA: 2.22E-05

TGF-beta receptor signaling	mRNA: 6.7E-04<br>DNA methylation: 0.01<br>miRNA: 0.01	Validated targets of C-MYC transcriptional activation	mRNA: 0.005<br>DNA methylation: 0.024<br>miRNA: 1.2E-07

Table S10: Top 2 pathways selected for each LSCC subtype and mean absolute SHAP values per pathway and omics type.

Subtype 1		Subtype 3	
Alpha6 beta4 integrin-ligand interactions	mRNA: 0.12<br>DNA methylation: 0.0027<br>miRNA: 0.006	Signaling mediated by p38-gamma and p38-delta	mRNA: 0.02<br>DNA methylation: 0.1<br>miRNA: 0.0006
Validated transcriptional targets of AP1 family members Fra1 and Fra2	mRNA:0.006<br>DNA methylation: 0.006<br>miRNA:0.029	CD40/CD40L signaling	mRNA: 0.0025<br>DNA methylation: 0.06<br>miRNA: 0.005
Subtype 2		Subtype 4	
IL2-mediated signaling events	mRNA: 0.005<br>DNA methylation: 0.003<br>miRNA: 0.04	ATM pathway	mRNA: 0.04<br>DNA methylation: 0.01<br>miRNA: 0.009
Role of Calcineurin-dependent NFAT signaling in lymphocytes	mRNA: 0.008<br>DNA methylation: 0.04<br>miRNA: 0.006	Regulation of Androgen receptor activity	mRNA: 0.003<br>DNA methylation: 0.025<br>miRNA: 0.02

Table S11: Top 2 pathways selected for each BRCA subtype and mean absolute SHAP values per pathway and omics type.

Subtype 1	Subtype 4

Canonical Wnt signaling pathway	mRNA: 6.8E-08<br>CNV: 0.001<br>miRNA: 4.7E-05	VEGFR3 signaling in lymphatic endothelium	mRNA: 6.5E-12<br>CNV: 0.002<br>miRNA: 3.8E-08
Stabilization and expansion of the E-cadherin adherens junction	mRNA: 2.1E-12<br>CNV: 0.0002<br>miRNA: 4E-06	CDC42 signaling events	mRNA: 0.0005<br>CNV: 0.008<br>miRNA: 0.0004
Subtype 2		Subtype 5	
Signaling mediated by p38-alpha and p38-beta	mRNA: 6.7E-05<br>CNV: 0.001<br>miRNA: 9.9E-06	a4b7 Integrin signaling	mRNA: 0.003<br>CNV: 0.04<br>miRNA: 0.028
TNF receptor signaling pathway	mRNA: 4.2E-06<br>CNV: 0.03<br>miRNA: 0.001	HIF-1-alpha transcription factor network	mRNA: 1.6E-05<br>CNV: 0.0001<br>miRNA: 5.4E-06
Subtype 3			
Neurotrophic factor-mediated Trk receptor signaling	mRNA: 1.96E-05<br>CNV: 0.002<br>miRNA: 4.3E-05		
Coregulation of Androgen receptor activity	mRNA: 0.0008<br>CNV: 0.01<br>miRNA: 0.0001		

6. Feature relevance in terms of SHAP values

Barplots of mean absolute SHAP values of omics features mapping to the most relevant cluster specific pathways according to Section 3 can be accessed under this link : https://docs.google.com/spreadsheets/d/11NWZRp2_DtyrvUhKQFRVq_KkDS41rzFpbQWoFU-1jSs/edit?usp=sharing

7. Mutational burden of most relevant pathways

CRC is missing, because none of the top 2 pathway genes contained somatic mutations.
The data underlying the Figures can be accessed via https://docs.google.com/spreadsheets/d/1FOJNR1sG86LUJ8dPWAk6POCQGrY1PhqgeZ5_u2ePvVE/edit#gid=1918286966

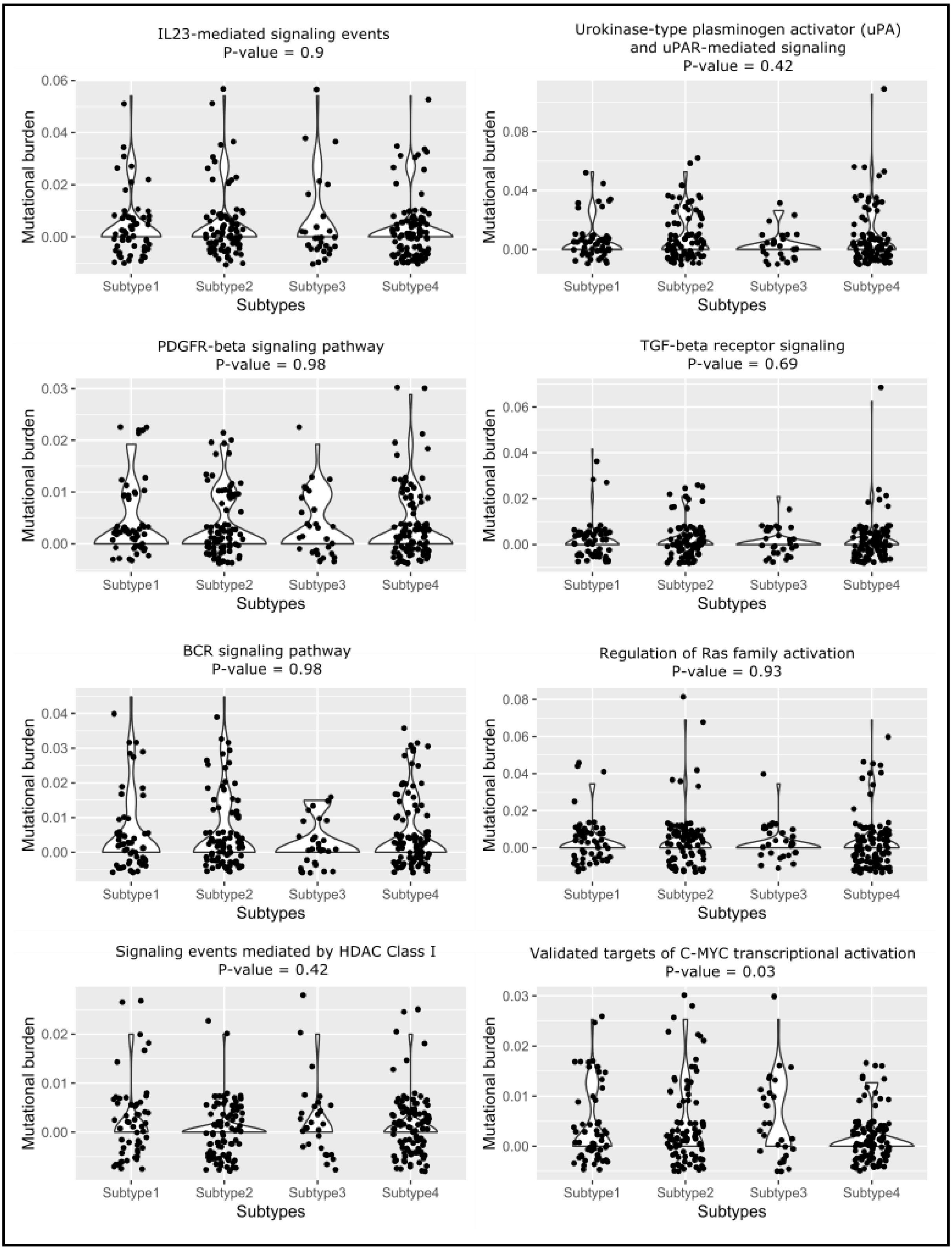

Fig S7: Mutational burden related to most relevant pathways across GBM subtypes.

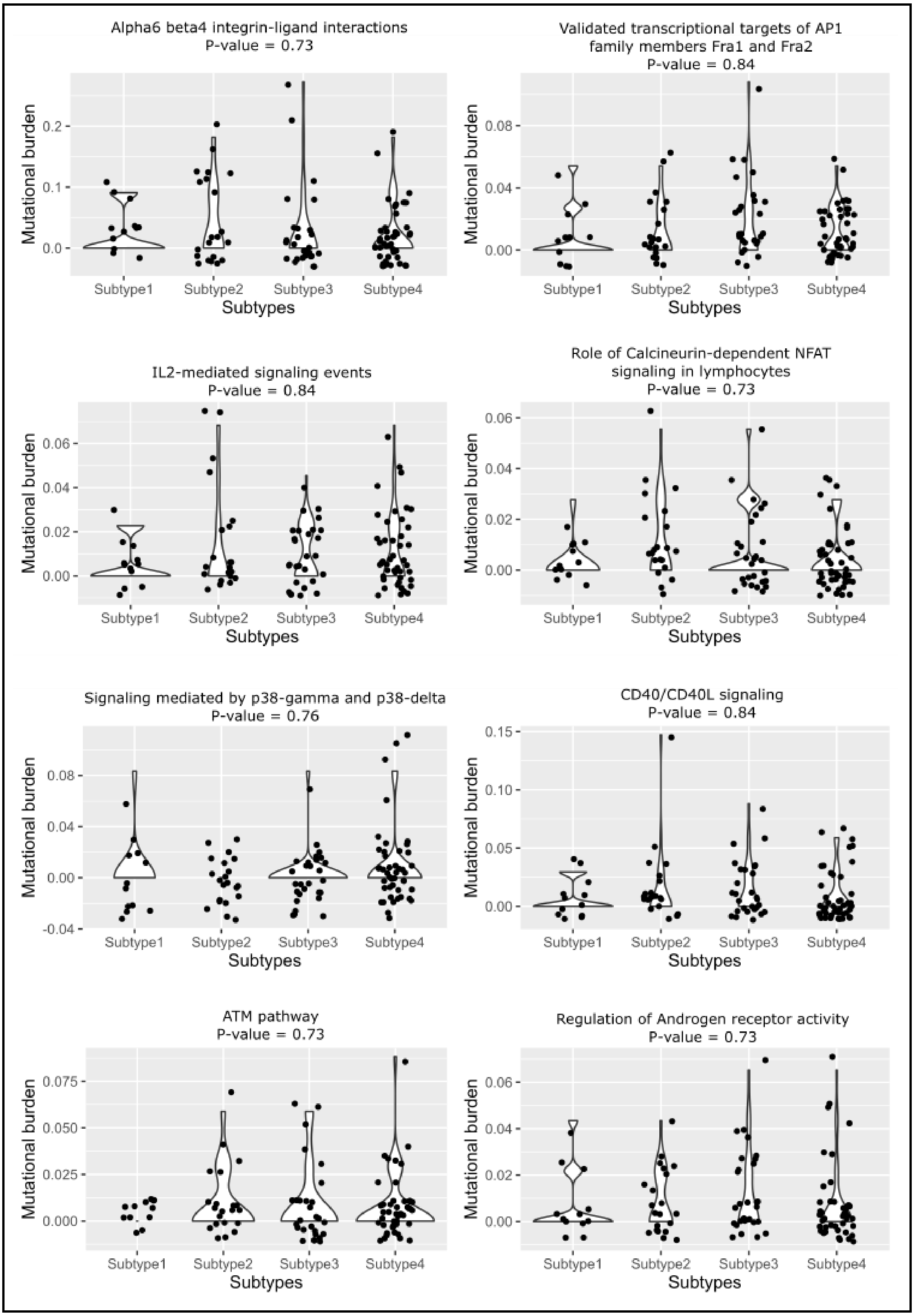

Fig S8: Mutational burden related to most relevant pathways across LSCC subtypes

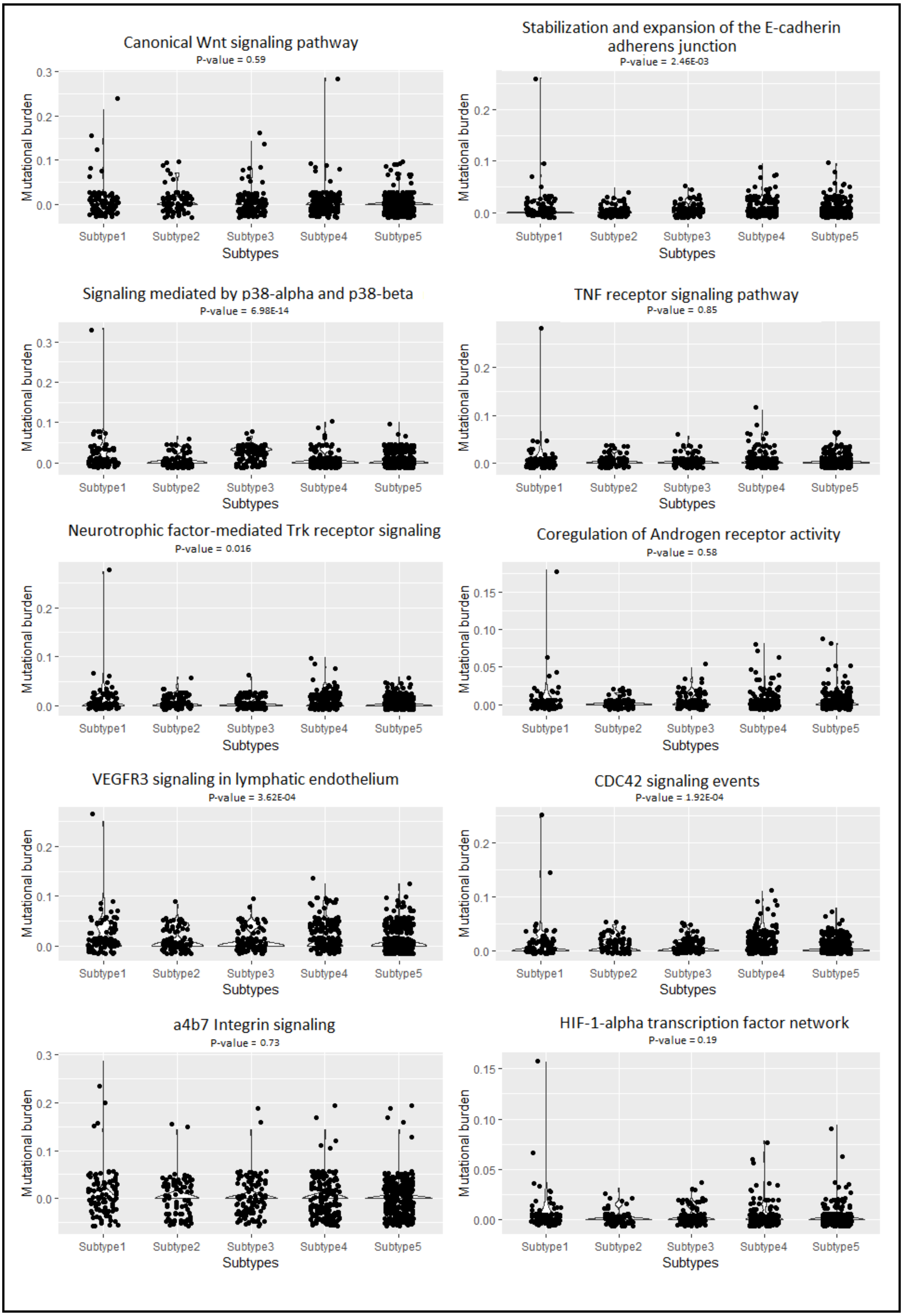

Fig S9: Mutational burden related to most relevant pathways across BRCA subtypes. The shown p-values have been corrected for multiple testing via the Benjamini & Hochberg method.

8. Loss curves from the multi-modal autoencoders

The Figures found under this weblink depict the autoencoder reconstruction loss as a function of training epochs. Due to the mass of Figures we restrict ourselves to the autoencoder models with optimal hyper-parameter settings (see main document for details about hyper-parameter tuning).

- CRC loss curves: https://gist.github.com/AminaLEM/dc0cbb124abbf5e3b096c31c95a04374
- GBM loss curves: https://gist.github.com/AminaLEM/6980bbe6fc6fda6242152ef491cc03ec
- LSCC loss curves: https://gist.github.com/AminaLEM/a70076ca5cf5d27e2edfa6df5c768251
- BRCA loss curves:https://gist.github.com/AminaLEM/6d6cae975486ecc573914b4aa8a28658

References

1. Benjamini Y, Hochberg Y (1995) Controlling the false discovery rate: a practical and powerful approach to multiple testing. Journal of the Royal statistical society: series B (Methodological) 57(1):289–300
