# Supplementary material for: PathME: pathway based multi-modal sparse autoencoders for clustering of patient-level multi-omics data
Source: BMC Bioinformatics. 2020 Apr 16;21:146. doi: 10.1186/s12859-020-3465-2 (PMC7161108; doi:10.1186/s12859-020-3465-2)
Supplement: Supplementary file 2 — Additional file 2. PathME code (python) and additional analysis codes: https://github.com/AminaLEM/PathME. [file 12859_2020_3465_MOESM2_ESM.docx]

<https://github.com/AminaLEM/PathME>.
